# A Nrf2-OSGIN1&2-HSP70 axis mediates cigarette smoke-induced endothelial detachment: implications for plaque erosion

**DOI:** 10.1093/cvr/cvad022

**Published:** 2023-02-18

**Authors:** Sandro Satta, Robert Beal, Rhys Smith, Xing Luo, Glenn R Ferris, Alex Langford-Smith, Jack Teasdale, Tom Tanjeko Ajime, Jef Serré, Georgina Hazell, Graciela Sala Newby, Jason L Johnson, Svitlana Kurinna, Martin J Humphries, Ghislaine Gayan-Ramirez, Peter Libby, Hans Degens, Bo Yu, Thomas Johnson, Yvonne Alexander, Haibo Jia, Andrew C Newby, Stephen J White

**Affiliations:** Department of Life Sciences, Manchester Metropolitan University, John Dalton Building, Chester Street, Manchester M1 5GD, UK; Department of Medicine, David Geffen School of Medicine, University of California, Los Angeles, CA 90095, USA; Department of Life Sciences, Manchester Metropolitan University, John Dalton Building, Chester Street, Manchester M1 5GD, UK; Department of Life Sciences, Manchester Metropolitan University, John Dalton Building, Chester Street, Manchester M1 5GD, UK; Department of Cardiology, The 2nd Affiliated Hospital of Harbin Medical University, & The Key Laboratory of Medical Ischemia, Chinese Ministry of Education, Harbin 150086, China; Department of Life Sciences, Manchester Metropolitan University, John Dalton Building, Chester Street, Manchester M1 5GD, UK; Department of Life Sciences, Manchester Metropolitan University, John Dalton Building, Chester Street, Manchester M1 5GD, UK; Bristol Medical School, Bristol Royal Infirmary, Upper Maudlin Street, Bristol BS2 8HW, UK; Laboratory of Respiratory Diseases and Thoracic Surgery, Department of Chronic Diseases and Metabolism, KU Leuven, Leuven, Belgium; Laboratory of Respiratory Diseases and Thoracic Surgery, Department of Chronic Diseases and Metabolism, KU Leuven, Leuven, Belgium; Bristol Medical School, Bristol Royal Infirmary, Upper Maudlin Street, Bristol BS2 8HW, UK; Bristol Medical School, Bristol Royal Infirmary, Upper Maudlin Street, Bristol BS2 8HW, UK; Bristol Medical School, Bristol Royal Infirmary, Upper Maudlin Street, Bristol BS2 8HW, UK; Wellcome Centre for Cell-Matrix Research, Faculty of Biology, Medicine & Health, University of Manchester, Manchester M13 9PT, UK; Wellcome Centre for Cell-Matrix Research, Faculty of Biology, Medicine & Health, University of Manchester, Manchester M13 9PT, UK; Laboratory of Respiratory Diseases and Thoracic Surgery, Department of Chronic Diseases and Metabolism, KU Leuven, Leuven, Belgium; Brigham and Women’s Hospital, Harvard Medical School, Boston, MA 02115, USA; Department of Life Sciences, Manchester Metropolitan University, John Dalton Building, Chester Street, Manchester M1 5GD, UK; Institute of Sport Science and Innovations, Lithuanian Sports University, Sporto g. 6, LT-44221 Kaunas, Lithuania; Department of Cardiology, The 2nd Affiliated Hospital of Harbin Medical University, & The Key Laboratory of Medical Ischemia, Chinese Ministry of Education, Harbin 150086, China; Department of Cardiology, Bristol Heart Institute, Upper Maudlin St., Bristol BS2 8HW, UK; Department of Life Sciences, Manchester Metropolitan University, John Dalton Building, Chester Street, Manchester M1 5GD, UK; Department of Cardiology, The 2nd Affiliated Hospital of Harbin Medical University, & The Key Laboratory of Medical Ischemia, Chinese Ministry of Education, Harbin 150086, China; Bristol Medical School, Bristol Royal Infirmary, Upper Maudlin Street, Bristol BS2 8HW, UK; Department of Life Sciences, Manchester Metropolitan University, John Dalton Building, Chester Street, Manchester M1 5GD, UK

**Keywords:** Endothelial erosion, Nrf2, adhesion, Autophagy

## Abstract

**Aims:**

Endothelial erosion of plaques is responsible for ∼30% of acute coronary syndromes (ACS). Smoking is a risk factor for plaque erosion, which most frequently occurs on the upstream surface of plaques where the endothelium experiences elevated shear stress. We sought to recreate these conditions *in vitro* to identify potential pathological mechanisms that might be of relevance to plaque erosion.

**Methods and results:**

Culturing human coronary artery endothelial cells (HCAECs) under elevated flow (shear stress of 7.5 Pa) and chronically exposing them to cigarette smoke extract (CSE) and tumour necrosis factor-alpha (TNFα) recapitulated a defect in HCAEC adhesion, which corresponded with augmented Nrf2-regulated gene expression. Pharmacological activation or adenoviral overexpression of Nrf2 triggered endothelial detachment, identifying Nrf2 as a mediator of endothelial detachment. Growth/Differentiation Factor-15 (GDF15) expression was elevated in this model, with protein expression elevated in the plasma of patients experiencing plaque erosion compared with plaque rupture. The expression of two Nrf2-regulated genes, OSGIN1 and OSGIN2, was increased by CSE and TNFα under elevated flow and was also elevated in the aortas of mice exposed to cigarette smoke *in vivo*. Knockdown of OSGIN1&2 inhibited Nrf2-induced cell detachment. Overexpression of OSGIN1&2 induced endothelial detachment and resulted in cell cycle arrest, induction of senescence, loss of focal adhesions and actin stress fibres, and disturbed proteostasis mediated in part by HSP70, restoration of which reduced HCAEC detachment. In ACS patients who smoked, blood concentrations of HSP70 were elevated in plaque erosion compared with plaque rupture.

**Conclusion:**

We identified a novel Nrf2-OSGIN1&2-HSP70 axis that regulates endothelial adhesion, elevated GDF15 and HSP70 as biomarkers for plaque erosion in patients who smoke, and two therapeutic targets that offer the potential for reducing the risk of plaque erosion.

## Graphical Abstract

**Graphical Abstract:**
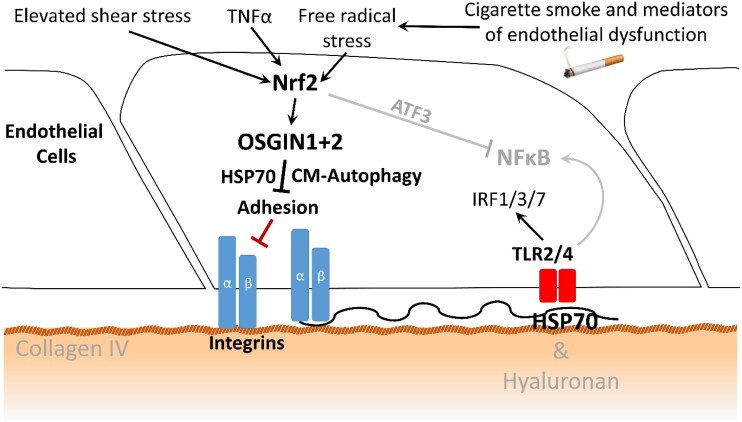
Elevated flow, cigarette smoke extract, and TNFα can trigger endothelial detachment, replicating features of plaque erosion in patients. Nrf2 activation or overexpression of OSGIN1&2 induces detachment of human coronary artery endothelial cells, likely through dysregulation of chaperone-mediated autophagy, that can be rescued by inhibition of HSP70 and 5′ AMP-activated protein kinase activation.

**See the editorial comment for this article ‘Smoke on the blood stream: novel insights in cigarette smoke-induced atherosclerosis and plaque erosion’, by H. Morawietz, https://doi.org/10.1093/cvr/cvad097.**

**Novelty and significance**

*What is known:*

- Plaque erosion is responsible for 25–30% of acute coronary syndromes
- Plaque erosion occurs on stenotic plaques where the endothelium is exposed to elevated flow
- Smoking is a common risk factor for plaque erosion

*What is new:*

- CSE and TNFαα cause endothelial detachment under elevated flow, with activation of Nrf2 contributing to, rather than protecting from, endothelial detachment
- OSGIN1&2 mediate Nrf2-dependent detachment, which can be abrogated by AMPK activation or HSP70 inhibition
- GDF15 and HSP70, identified by our model, are elevated in patients who experience plaque erosion compared with those who experience plaque rupture

## 1. Introduction

Erosion of the endothelium overlying stenotic plaques causes about a third of atherothrombotic acute coronary syndromes (ACS), most of the remainder result from plaque rupture.^1–3^ Patients who experience plaque rupture or plaque erosion have different demographics and risk factor profiles indicating a divergence in the mechanism driving ACS. Older patients with diabetes, hyperlipidaemia, or hypertension have elevated risk of plaque rupture, whereas a higher frequency of plaque erosion is observed in smokers and patients who are younger and/or female.^4–9^ Similarly, plaques that rupture or erode have different histological features. For example, plaque rupture commonly complicates macrophage and lipid-rich plaques with thin fibrous caps. In contrast, sites of erosion generally occur on plaques containing many smooth muscle cells and few resident leucocytes with an intimal extracellular matrix rich in versican and hyaluronan, molecules implicated in altering endothelial function, through engagement of different integrins, and cellular receptors.^1,10–12^

The mechanism that evokes plaque erosion remains enigmatic; however, several studies identified a high prevalence of smoking in plaque erosion patients, which may offer insight into the underlying mechanism.^4,6,9,13,14^ Smokers have elevated circulating mediators of inflammation, such as tumour necrosis factor-alpha (TNFα), that exacerbate endothelial dysfunction.^15^ Additionally, cigarette smoke contains abundant oxidants and free radicals,^16,17^ demonstrated to activate the antioxidant defence regulated by Nrf2.^18^ Smoking increases the expression of Nrf2-target genes in the lung,^19^ which may reduce smoking-induced damage.^20^ We previously demonstrated that fresh aqueous cigarette smoke extract (CSE) also activates Nrf2 in human coronary artery endothelial cells (HCAECs).^21,22^

The haemodynamic environment modifies many aspects of endothelial behaviour.^23–25^ Consequently, low average shear stress and disturbed flow promote plaque development and progression;^26,27^ in contrast, both plaque erosion and plaque rupture most frequently occur on the upstream surface of stenotic plaques where the endothelium encounters elevated flow.^4,11,28–32^ We previously demonstrated that elevated flow modifies endothelial behaviour by eliciting a specific pattern of gene expression, amplifying the antioxidant-stress response and increasing the expression of transcription factor ATF3 that suppresses the expression of many NFκB-regulated genes.^21,33^ However, the consequences of these gene expression changes for endothelial adherence to the substrate and detachment were not previously investigated. Herein, we describe that the combination of cigarette smoke and inflammation induced endothelial cell detachment, identifying a key role for Nrf2 and for two Nrf2-regulated genes, OSGIN1 and OSGIN2 in this process, referencing our results with analysis within the vasculature of mice exposed to cigarette smoke and measurements in patients experiencing plaque rupture or plaque erosion.

## 2. Methods

An expanded and detailed methods section is supplied in the supplementary data file.

### 2.1 Tissue culture

HCAECs were obtained from Promocell and used between passages 4 and 6. HCAECs were seeded on 0.1% gelatin-coated slides with a density of 2.5 × 10^5^ and cultured for a minimum of 3 days to ensure complete confluency, production, and reorganization of sub-cellular matrix and maturation of cell–cell junctions. Culture under flow was performed using a parallel plate flow apparatus as described.^21,33,34^ For single gene overexpression studies, 200 pfu/cell active vector (AdOSGIN1, AdOSGIN2, or AdNrf2) was combined with 200 pfu/cell of control virus to make a total of 400 pfu/cell; for dual gene overexpression, 200 pfu/cell of both vectors were added; 400 pfu/cell of Adcontrol, E1/E3 ‘empty’ Ad vector, was used as the control.

### 2.2 Patient study population and blood samples

Patients presenting with ST-segment elevation myocardial infarction who underwent primary percutaneous coronary intervention (PCI) at the Second Affiliated Hospital of Harbin Medical University were prospectively enrolled in this study. Patients were divided into plaque erosion and plaque rupture group according to optical coherence tomography (OCT) imaging of the culprit lesion. The plaque rupture and plaque erosion were defined by OCT based on our previously established definition.^35^ The blood samples around the culprit lesion were collected from intracoronary aspirates during the primary PCI. Written informed consent was obtained from all patients. This study was approved by the institutional research ethics committee of the Second Affiliated Hospital of Harbin Medical University and conforms to the principles outlined in the Declaration of Helsinki.

### 2.3 RNASeq data analysis on HCAEC with OSGIN1&2 overexpression

Strand-specific RNAseq libraries were prepared using the Illumina workflow with the TruSeq® Stranded mRNA Sample Preparation Kit. Paired-end reads were generated on the Illumina platform of HiSeq4000. The predicted upstream regulators and altered canonical pathways were generated by IPA (QIAGEN Inc.).^36^ The cluster analysis was carried out on the differentially expressed genes identified with DESeq2 using a *P*-adjusted cut-off of 0.05, an absolute log2 fold change cut-off of 0.5, and a base mean cut-off of 50. The Pearson distance was clustered with the hclust function and plotted with an R package of gplots.

### 2.4 Immunofluorescence on mouse aortas

Frozen sections of aortas from mice exposed to air (control mice) or cigarette smoke for 3 months (as described^37^) were analysed by immunofluorescence. The mouse tissue used here was surplus to the described study.^37^ All experimental procedures were carried out in accordance with relevant guidelines and regulations and approved by the Ethics Committee of Animal Experiments of the KU Leuven.

### 2.5 Statistical analysis

Data are presented as means ± standard error of the mean (SEM) of at least three independent experiments, with different donors used for each *n*. One-way analysis of variance (ANOVA) was used to determine the difference between three or more groups. Two-way ANOVA was used to evaluate three or more groups on different conditions. *P* < 0.05 were considered to indicate statistically significant differences. GraphPad Prism software was used for statistical analysis (GraphPad Software, La Jolla, CA).

## 3. Results

### 3.1 Elevated flow with simulated smoking triggers endothelial cell detachment

**Figure 1:**
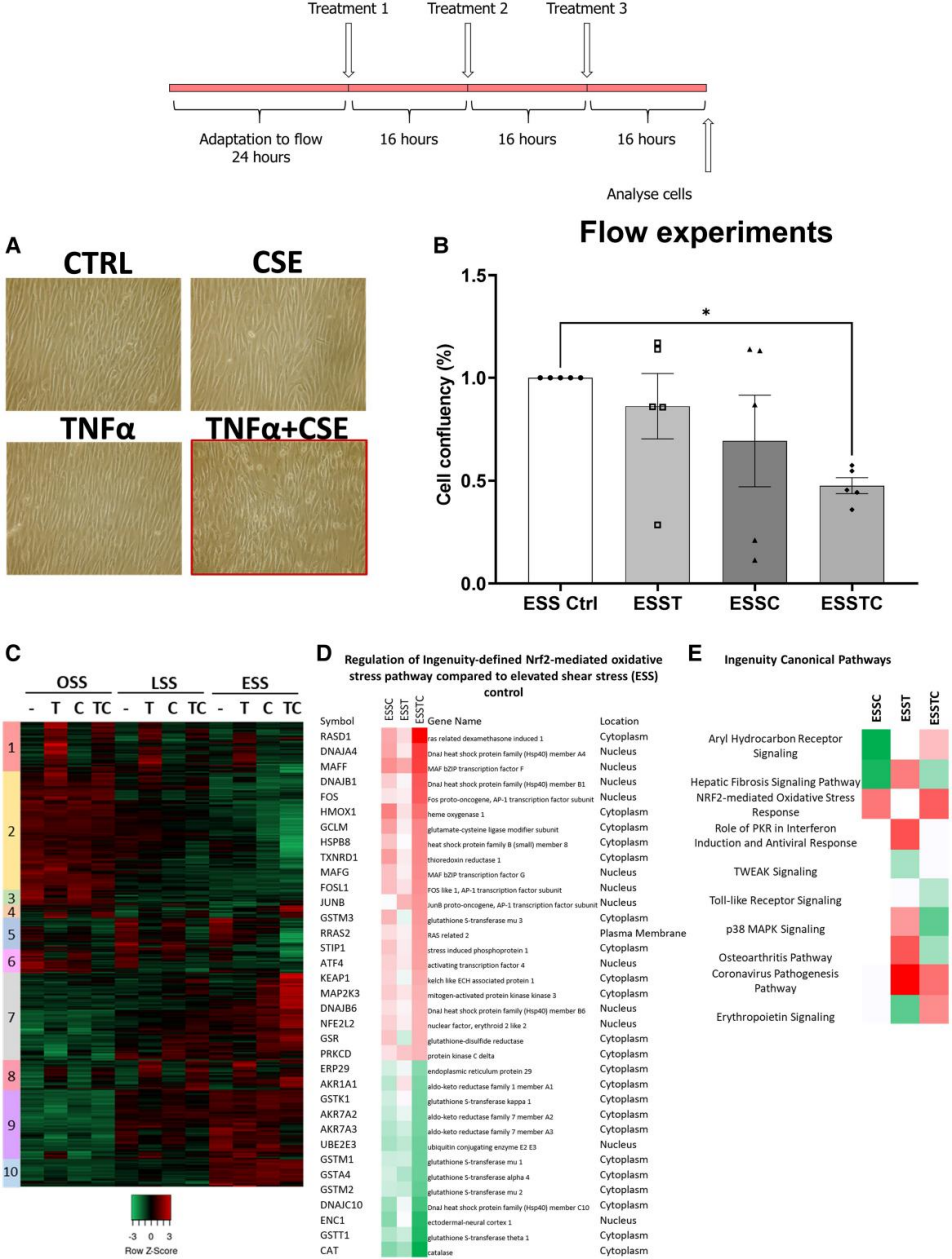
*In vitro* modelling of the conditions found in endothelial erosion. (*A*) Photomicrographs of HCAECs, cultured under elevated flow for 72 h (7.5 Pa), with the experimental design of the *in vitro* model illustrated above. (*B*) Quantification of detachment in HCAECS cultured under elevated flow with the addition of TNFα (T-5 ng/mL) and CSE (C-10%) or both (TC), with the combined treatment resulting in a significant loss of cell adhesion (*P < 0.05, *n* = 6 donors). (*C*) Heatmap with identified clusters of gene expression changes identified in the transcriptomic analysis of the combined effects of oscillatory (OSS), laminar (LSS), and elevated (ESS) shear stress on HCAECs treated with control, 3 doses of 5 ng/mL TNFα (T), 3 doses of 10% CSE (C), or the combination of TNFα and CSE (TC), *n* = 3 donors. (*D*) Regulation of Ingenuity-defined Nrf2-mediated oxidative stress pathway compared with ESS control. (*E*) Top predicted canonical pathways by Ingenuity analysis, red indicates a predicted activation and green a decrease, with white suggesting dysregulation.

We and others have demonstrated that plaque erosion most frequently occurs on modestly stenotic plaques where the endothelium is exposed to elevated flow.^29–32^ Therefore, we compared the response of HCAECs cultured under oscillatory (OSS: ±0.5 Pa, 1 Hz, athero-prone), normal laminar (LSS: 1.5 Pa, athero-protective), or pathologically relevant elevated laminar shear stress (ESS: 7.5 Pa, erosion-prone) to simulated smoking using aqueous 10% CSE with or without 5 ng/mL TNFα to model smoking-induced lung inflammation. ESS at 7.5 Pa approximates to a modestly stenotic plaque with ∼40% diameter stenosis, which aligns with the median stenosis observed in patients with OCT-defined plaque erosion.^29–32^ To allow HCAEC adaptation and alignment to the flow environment, confluent monolayers of HCAECs were cultured under flow for 24 h prior to any treatment. Subsequently, three boluses of 10% fresh aqueous CSE and/or 5 ng/mL TNFα were injected into the flow system at 16 h intervals to simulate the sustained endothelial dysfunction experienced by smokers. We have previously described concentration dependence, analysis of cell viability, and temporal regulation of gene expression by these treatments, showing that 16 h intervals sustain Nrf2- and TNFα-driven gene expression.^21,22^ We report here that reproducible cell detachment occurred only under elevated flow and after the addition of both CSE and TNFα (∼50% cell loss; *Figure**1A* and *B*). Transcriptomic analysis revealed the response to CSE, TNFα, and the combination of CSE and TNFα depended on the flow environment (*Figure 1C* and Supplementary material online, *Figure S1*), regulating a distinct subset of genes. As expected, given its pro-oxidant effects, CSE increases Nrf2-dependent gene expression in cultured HCAECs, an antioxidant response that is generally considered protective^21,38^ (*Figure**1D* and *E*).

### 3.2 Investigation of the pathways responsible for HCAEC detachment

A range of pharmacological agents were investigated to test their ability to prevent CSE and TNFα-dependent HCAEC detachment under elevated flow. Co-treatment with an apoptosis inhibitor (20 µM Z-VAD-FMK) did not prevent cell detachment, consistent with our previous observations that CSE did not induce apoptosis^22^ (see Supplementary material online, *Figure S2*). Similarly, co-treatment with pan-matrix metalloproteinase inhibitor (10 µM GM6001), treatment with a statin (3 µM rosuvastatin), antioxidant (200 µM apocynin), or necrosis inhibitor (10 µM necrostatin-1) all failed to prevent cell detachment (see Supplementary material online, *Figures S3–S6*).

### 3.3 Nrf2 participates in elevated flow plus CSE plus TNFα-induced endothelial cell detachment

**Figure 2:**
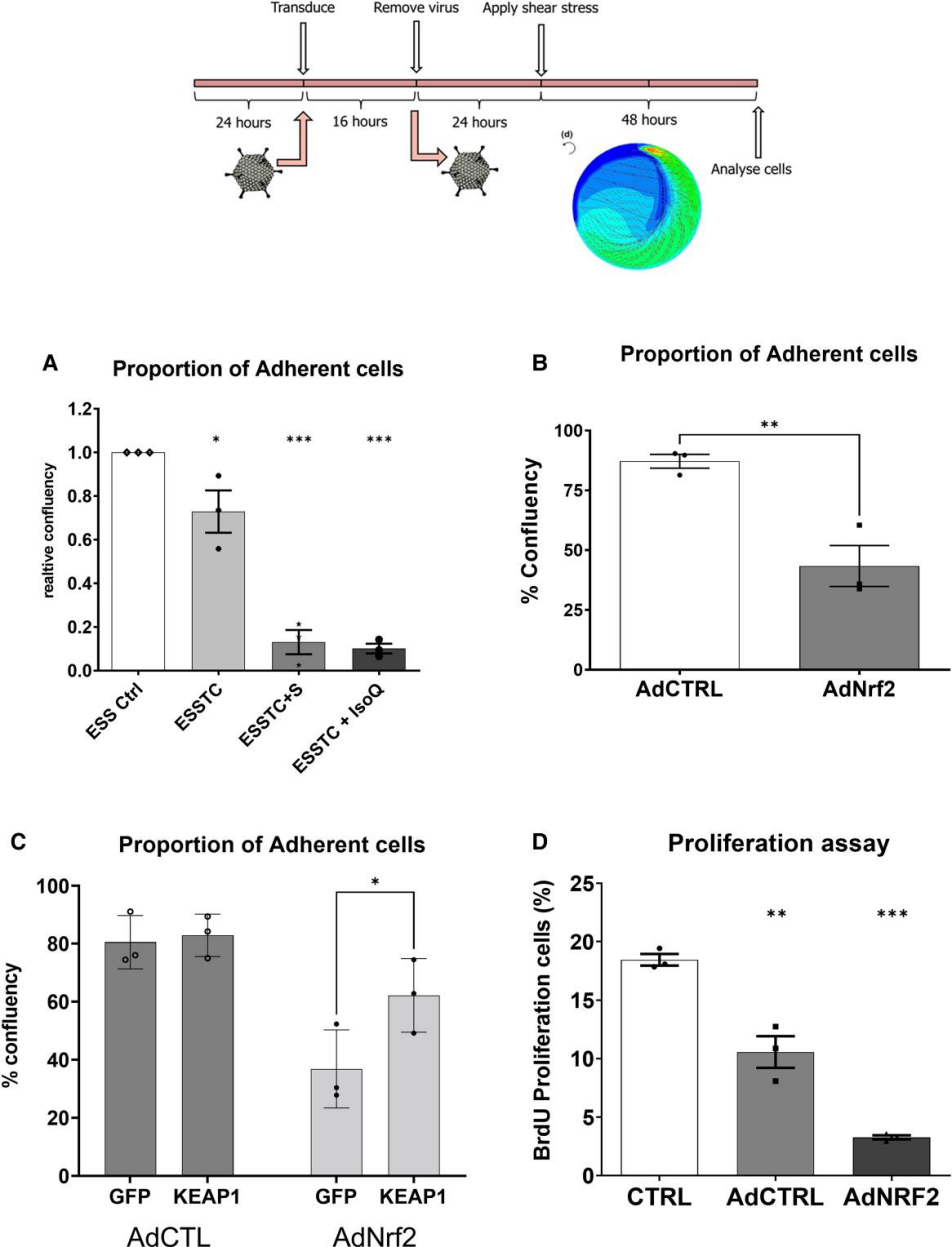
Evidence for a role for Nrf2 in endothelial detachment. (*A*) Quantification of cell number in elevated laminar shear stress control (ESS), with the addition of TNFα and CSE [mean ± SD, one-way ANOVA, elevated flow + TNFα + CSE (ESSTC), 30% reduction vs. ESS, *P* < 0.05, *n* = 3], and Nrf2 activator sulforaphane (ESSTC-S, 2.5 μM, 5-fold reduced adhesion vs. ESSTC, *P* < 0.05, *n* = 3) or isoliquiritigenin (ESS-IsoQ, 10 μM, 9-fold reduced adhesion vs. ESSTC, *P* < 0.05, *n* = 3). (*B*) Adenoviral overexpression of Nrf2 (200 pfu/cell and 200 pfu/cell AdCTRL combined to match later experiments) promotes 50% of cell detachment compared with AdCTRL (400 pfu/cell) (****P < 0.0001, *n* = 3) using the experimental design illustrated above. (*C*) Transduction with lentiviral control (GFP) or lentiKEAP1 prior to adenoviral overexpression of Nrf2 (as C) resulted in a significant reduction of Nrf2-dependent detachment (*P < 0.05, *n* = 3). (*D*) BrdU proliferation assay in HCAECs treated with adenoviral overexpression of wild-type Nrf2, % BrdU positive cells, mean and SEM obtained from *n* = 6, *P < 0.05 and **P < 0.01, compared with control.

We hypothesized that further activation of Nrf2 might protect against the effects of CSE and TNFα treatment and prevent HCAEC detachment through amplification of the antioxidant response. Unexpectedly, the addition of two different pharmacological activators of Nrf2 (2.5μM sulforaphane or 10μM isoliquiritigenin) to the cultures with elevated flow, CSE and TNFα, triggered almost complete cell detachment (*Figure 2A*), suggesting that chronic hyperactivation of Nrf2 contributes to, rather than protects from, cell detachment. We tested this using adenoviral overexpression of Nrf2 in HCAECs, exposing them to shear stress using an orbital shaker.^39^ This exposes HCAECs to laminar flow at the periphery of the well and oscillatory flow at the centre, standardizing the mechanical forces to which the HCAECs are exposed across experiments. Overexpression of Nrf2 triggered cell detachment (*Figure 2B*), which was reduced by lentiviral overexpression of the Nrf2 inhibitor KEAP1 (*Figure 2C*), confirming a role for Nrf2-regulated gene expression in promoting HCAEC detachment. In addition, we observed that Nrf2 overexpression significantly inhibited HCAEC proliferation (*Figure 2D*).

### 3.4 CSE, TNFα, and elevated flow augment OSGIN1 and OSGIN2 expression in HCAECs

**Figure 3:**
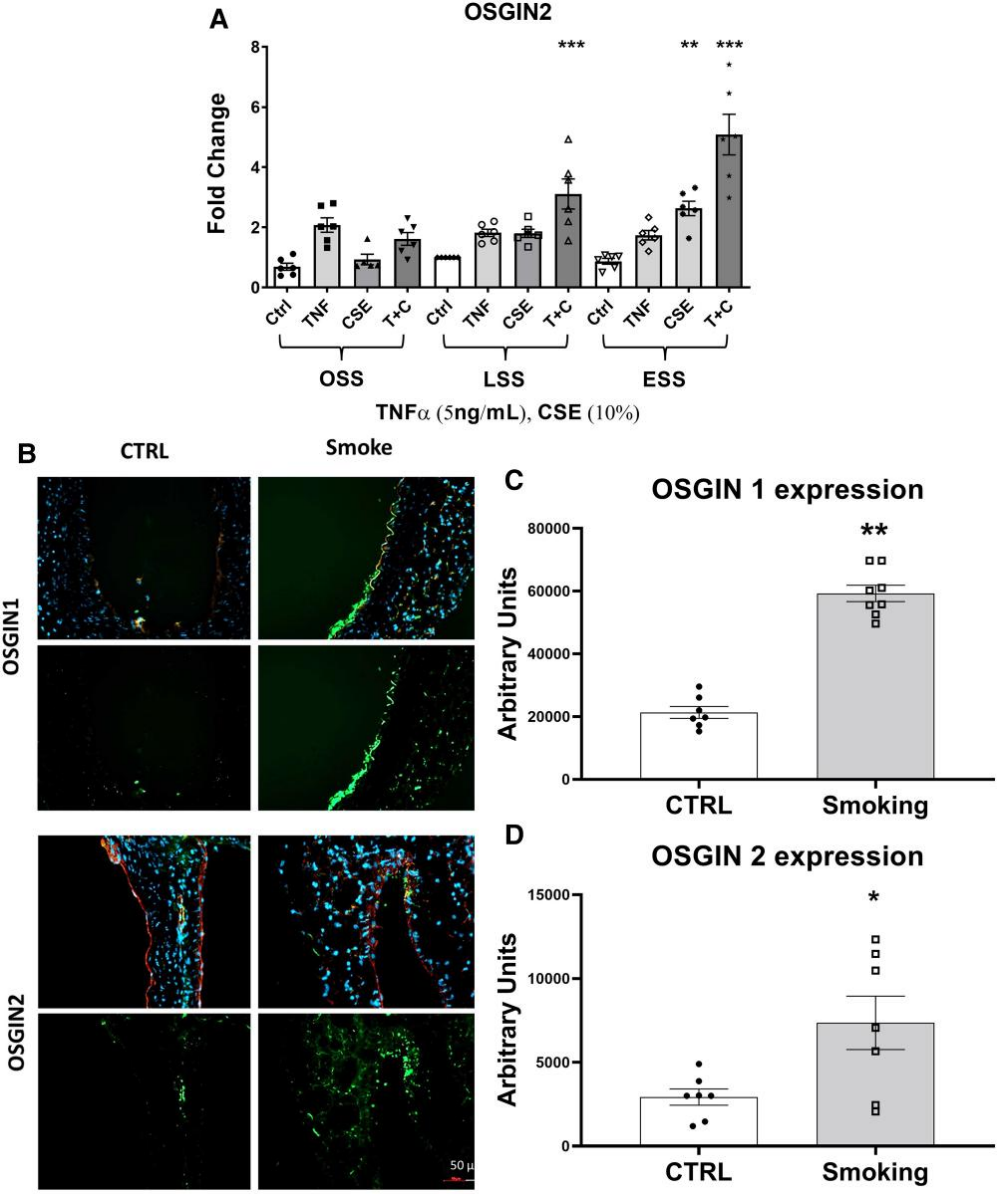
Regulation of OSGIN1&2 by cigarette smoke. (*A*) OSGIN2 mRNA expression in HCAECs cultured under OSS, LSS, or ESS, with TNFα (5 ng/mL) or CSE (10%) or both (*P = 0.05, **P < 0.01, ***P < 0.001 vs. LSS CTRL, mean, and SEM, *n* = 6, two-way ANOVA). (*B*) Immunohistochemical staining on 8 µm sections of aortas from male mice exposed to cigarette smoke for 3 months with quantification of staining; green: OSGIN1 or OSGIN2; red: CD31; and blue: DAPI nuclear stain. (*C* and *D*) Quantification of immunofluorescence staining (**P < 0.01, *n* = 8, OSGIN1 vs. 7 CTRL; *P < 0.05, *n* = 7, OSGIN2 vs. 7 CTRL, mean, and SEM).

We previously reported that Nrf2 regulates the expression of oxidative stress growth inhibitor 1 (OSGIN1) in HCAECs,^22^ and that OSGIN1 rises along with a panel of other Nrf2-regulated genes by ESS, TNFα, and CSE.^21^ Here we investigated the expression of OSGIN2, under the same conditions as both OSGIN1 and OSGIN2 were increased in the transcriptomic analysis in ESSTC compared with ESS. Elevated flow + CSE + TNFα treatment (ESSTC) resulted in the highest level of expression of OSGIN2 (*Figure 3A*) and broadly mirrors that of OSGIN1 (reproduced in Supplementary material online, *Figure S3*). To investigate whether exposure to cigarette smoke increased the expression of OSGIN1 and OSGIN2 in vascular tissue *in vivo*, we performed immunohistochemical analyses on the aortas of mice exposed to cigarette smoke for 3 months.^37^ We observed significantly higher OSGIN1&2 protein expression with prominent staining of OSGIN1 in the luminal endothelial cells and widespread expression of OSGIN2 throughout the vessel wall in the aortas of mice exposed to cigarette smoke compared with control (*Figure 3B–D*).

### 3.5 OSGIN1 and OSGIN2 overexpression inhibits human coronary artery endothelial cell proliferation and induces senescence

**Figure 4:**
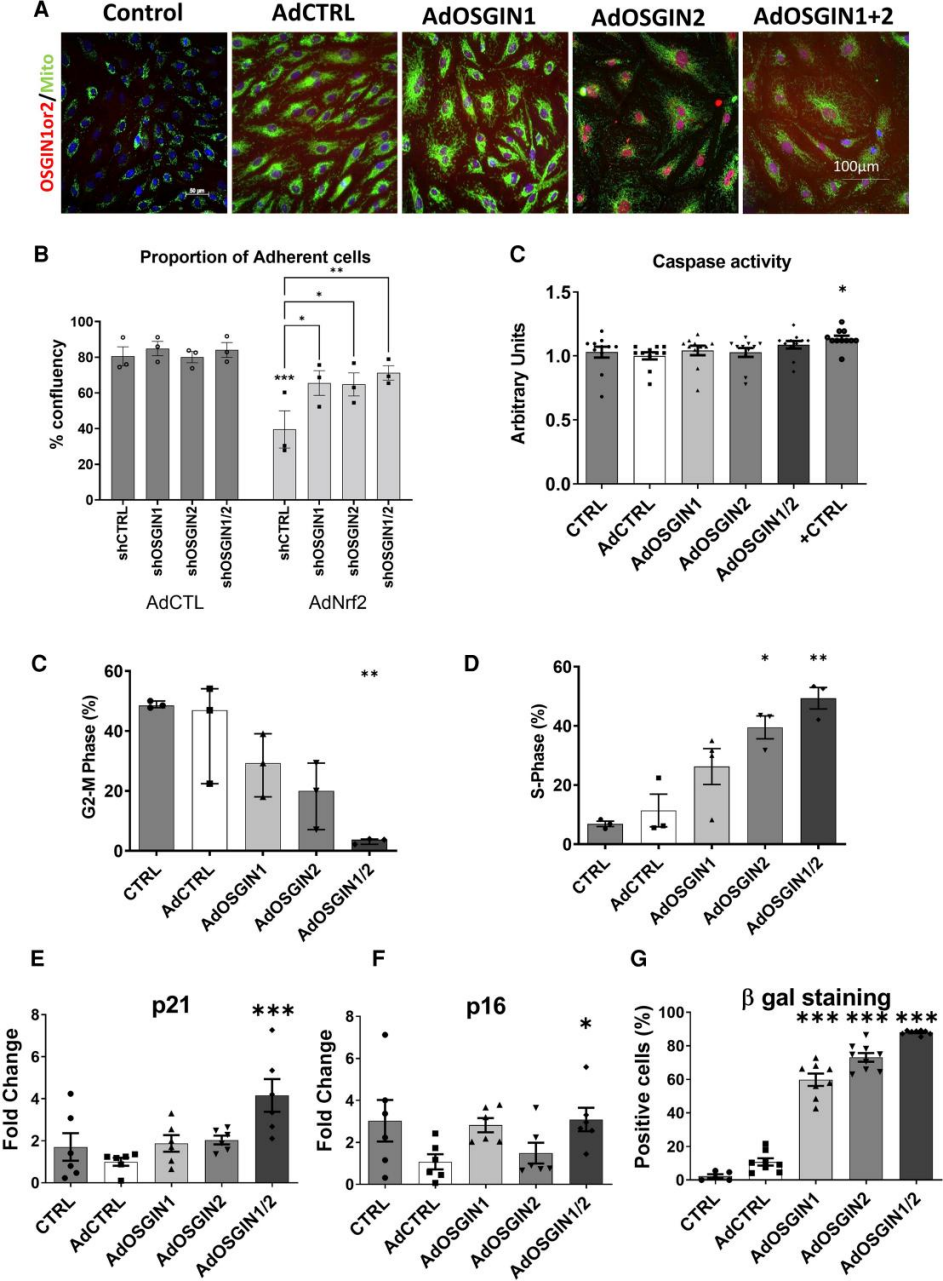
OSGIN1&2 overexpression effects endothelial proliferation and senescence. (*A*) Immunocytochemical staining for OSGIN1 and OSGIN2 (in red) shows their nuclear localization. Mitochondrial staining (in green) confirmed no localization of OSGIN1 and OSGIN2 (scale bar 100 µm). (*B*) Quantification of adhesion upon transduction with lentiviral shCtrl or lentiviral vectors overexpressing short hairpin targeted against OSGIN1 (shOSGIN1), OSGIN2 (shOSGIN2), or both vectors (shOSGIN1/2) with subsequent transduction with AdCtrl or AdNrf2 as described in *Figure 2C*). (*C*) Caspase 3/7 activity of HCAECs transfected with adenovirus overexpressing OSGIN1, OSGIN 2, and both together, compared with AdCtrl transfections and positive control (0.2 mM H2O2). The caspase 3/7 activity was measured using Caspase-Glo 3/7 assay. Data are presented as mean ± SEM (*n* = 6). **P < 0.01. (*D* and *E*) Flow cytometry analysis after adenoviral-mediated overexpression of OSGIN1 and OSGIN2 showed inhibition of cell cycle in HCAECs, with cells accumulating in S-phase, without proceeding to division (mean ± SD, one-way ANOVA, *P < 0.05, and ***P < 0.001, *n* = 3). (*F* and *G*) Changes in mRNA expression of p-21^Waf-1^ and p-16^INK4a^ with OSGIN1, and/or 2 overexpression (***P < 0.001; *P < 0.05 vs. AdCTRL, mean ± SEM, *n* = 6). (*H*) Increased staining for senescence-associated β-galactosidase and up-regulation of p-21^Waf-1^ and p-16^INK4a^ demonstrates the induction of the senescence pathway and lysosomal accumulation by OSGIN1&2 (***P < 0.001, median and inter-quartiles, *n* = 3; Supplementary material online, *Figure S16*).

The function of OSGIN1&2 in vascular tissues has not been previously investigated; hence, we investigated adenoviral overexpression of OSGIN1 and OSGIN2 in HCAECs. OSGIN1&2 have a similar predicted tertiary structure (40.8% amino acid sequence similarity) and demonstrate high conservation across species and contain functional Nrf2 binding sites in their promoters (see Supplementary material online, *Figures S7*). OSGIN1&2 localized to the nucleus (*Figure 4A*), despite the lack of identifiable nuclear localization sequences.

We tested if OSGIN1 and/or OSGIN2 participated in the Nrf2-dependent defect in HCAEC adhesion, shRNA knockdown of OSGIN1&2 reduced Nrf2-driven cell detachment (*Figure 4B*), indicating their involvement in this process. Overexpression of OSGIN1 or 2 did not induce apoptosis (*Figure 4C* and Supplementary material online, *Figure S8*); however, similar to Nrf2, overexpression of OSGIN1&2 inhibited proliferation of HCAECs, with cells accumulating in S-phase and not proceeding to cytokinesis (*Figure 4C–D*). Large multi-nucleated cells accumulated (Supplementary material online, *Figures S9–S11*, and observable in *Figure 6A–C*) corresponding to an increase in senescence-associated β-galactosidase staining and increased expression of cyclin-dependent kinase inhibitors p-16^INK4a^ (CDKN2A) and p-21^Waf-1^ (CDKN1A) (*Figure 4E–G*), all consistent with cells undergoing senescence.

### 3.6 OSGIN1 and OSGIN2 regulate human coronary artery endothelial gene expression

**Figure 5:**
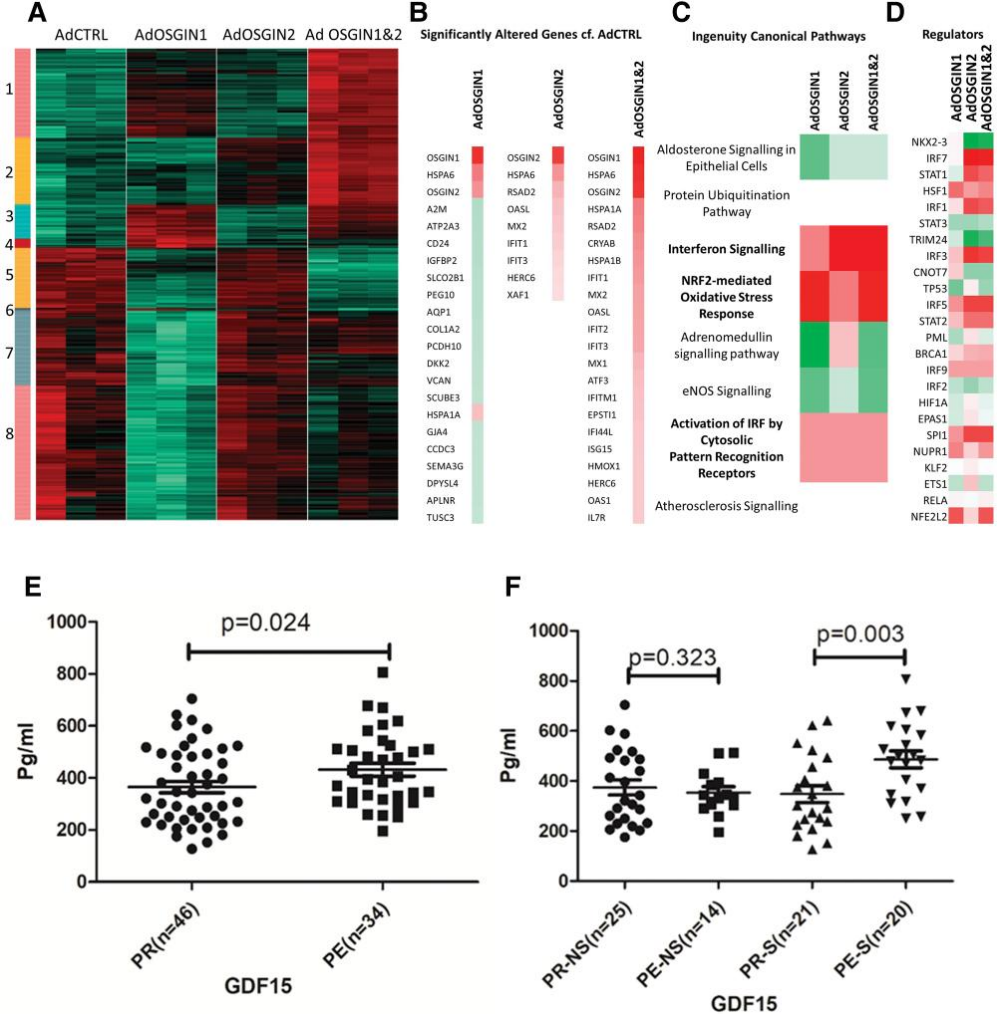
Changes in gene expression in HCAECs overexpressing OSGIN1, 2, or 1&2 and analysis of GDF15 expression in ACS patients. (*A*) Heatmap of gene expression significantly changed between AdCTRL or AdOSGIN1, 2, or 1&2 (*n* = 3); green indicated decreased, black no change, and red increased gene expression. Clustering analysis revealed eight clusters. (*B*) The genes with the most significant/largest fold change by AdOSGIN1, 2, or 1&2 compared with AdCTRL. Red indicates an increase in expression and green a decrease. (*C*) Predicted upstream transcriptional regulators; red indicates a predicted activation, white no change, and green a decrease. (*D*) Top predicted canonical pathways; red indicates a predicted activation and green a decrease, with white suggesting dysregulation. (*E*) Quantification of circulating GDF15 levels in serum from patients with OCT-defined plaque rupture (PR) and OCT-defined plaque erosion (PE). (*F*) Subgroup analysis of circulating GDF15 levels in smokers (S) and non-smokers (NS), with OCT-defined PR and OCT-defined PE.

RNASeq was performed on HCAECs transduced with adenoviral control (AdCTRL), AdOSGIN1, AdOSGIN2, or AdOSGIN1 & 2 (*Figure 5A–D*) under static culture conditions. The relative expression of 360 genes were differentially regulated between these conditions (*P*-adj < 0.05). Cluster analysis identified eight clusters (*Figure 5A*); of note, Cluster 1 (genes activated strongly by OSGIN1 and the combination of OSGIN1&2) was enriched in genes associated with the Nrf2-mediated oxidative stress response, inhibition in eNOS signalling, and alteration of the unfolded protein response (for full list, see Supplementary material online, *Table S5*). These changes likely reflect the activation of the transcription factors HSF1 and NFE2L2 (Nrf2) (see Supplementary material online, *Table S5*). Cluster 2 (genes strongly up-regulated by OSGIN2 and OSGIN1&2) associates with the activation of interferon signalling (predominantly type I) typically driven by the activation of STAT1 and 2, IRF1, 3, 5, 7, and 9, and inhibition of TRIM24 (see Supplementary material online, *Table S6*). The Nrf2-mediated Oxidative Stress Response pathway driven by Nrf2 activation dominated Cluster 3 (genes up-regulated by OSGIN1, Supplementary material online, *Table S7*, see full details of all other clusters in Supplementary material online, *Tables S8–*S15).

With AdOSGIN1, 235 genes differed significantly (*P*-adj < 0.05), 9 with AdOSGIN2 and 169 with AdOSGIN1 + 2 compared with control (*Figure 5B*). Of the top 20 genes whose expression rose with OSGIN1&2 expression, 11 participate in interferon (or TLR2/4) signalling or lie downstream of interferon (*Figure 5B*) with predicted upstream regulators IRF7, STAT1, IRF1, IRF3, IRF5, STAT2, BRACA1, IRF9, and SPI1 (*Figure 5D*). Six are associated with proteostasis; five of these interact with the key proteostasis regulator BAG3, which co-ordinates chaperone-mediated autophagy (HSPA6, HSPA1A, CRYAB, HSPA1B, and ISG15; Ingenuity IPA database). Furthermore, other interaction partners of BAG3: STIP1, HSPB1, HSPB8, NQO1, HSP90AA1, HSP90AB1, DNAJB1, DNAJB6 HSPA4, P4HA2, SQSTM1 (p62), HSPA8, TRIM69, all increase with OSGIN1&2 overexpression (see Supplementary material online, *Table S15*). In summary, the transcriptomic analysis of OSGIN1&2 overexpression identifies an increase in interferon/toll-like receptor (TLR) signalling, Nrf2 and HSF1-regulated gene expression, and pronounced changes in genes involved in BAG3-regulated proteostasis.

Analysis of the genes regulated by both ESSTC and OSGIN1&2 overexpression was selected using Venny 3.0; 110 genes were co-regulated by the 2 conditions (see Supplementary material online, *Figure S14*). The complete list of the overlapping genes is summarized in Supplementary material online, *Table S16*. To further evaluate gene–gene interaction, STRING network and Cytoscape neighbour analysis was performed. Of interest, Growth/Differentiation Factor-15 (GDF15), a known biomarker of cardiovascular risk,^40–42^ was increased by elevated flow with the addition of TNFα and CSE compared with elevated flow control (five-fold, adj *P* < 0.001; Supplementary material online, *Figure S15A*) and OSGIN1&2 overexpression. We, therefore, quantified the level of GDF15 in the plasma of patients with OCT-defined plaque erosion compared with OCT-defined plaque rupture. GDF15 was significantly elevated in plaque erosion compared with plaque rupture patients (*Figure 5E*; 433 ± 141, *n* = 34 vs. 365 ± 148 pg/mL, *n* = 46, *P* = 0.024). This relationship was driven by smoking, with no differences observed between plaque rupture or plaque erosion patients who were non-smokers, whereas a significant difference was observed between erosion and rupture patients who smoked (*Figure 5F*; 487 ± 146, *n* = 21 vs. 348 ± 149 pg/mL, *n* = 20, *P* = 0.003). The overall effect of smoking, independent of ACS type, did not reach statistical significance in this cohort (Supplementary material online, *Figure S19B*, *n* = 41 vs. *n* = 39, *P* = 0.074).

### 3.7 OSGIN1 and OSGIN2 overexpression dysregulate the cytoskeleton, focal adhesions, and autophagy

**Figure 6:**
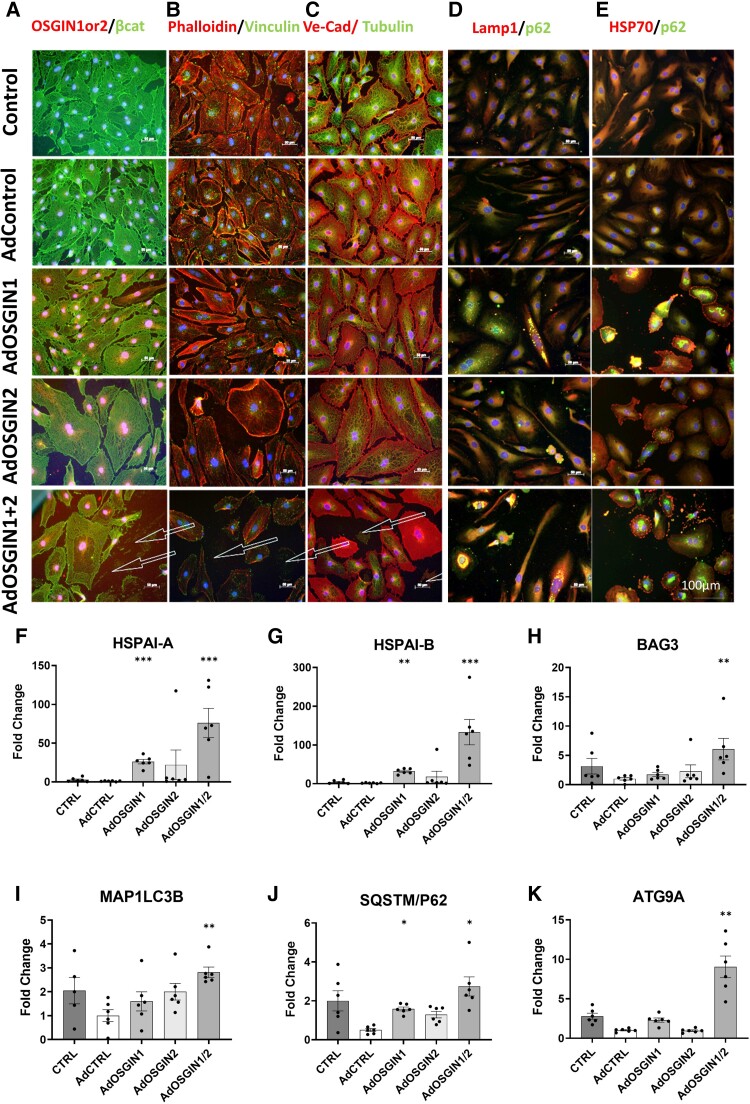
OSGIN1&2 effects on cell structure and autophagy-related gene expression. (*A–C*) Immunocytochemical analysis of HCAECs with adenoviral-mediated overexpression of OSGIN 1 + 2 (scale bar 100 µm). β-Catenin (a marker of intercellular junctional stability), vinculin (focal adhesions) and phalloidin (Actin), Tubulin and VE-Cadherin (intercellular junctions in red), visualized by immunofluorescence microscopy. OSGIN1 + 2 overexpression profoundly affects cell structure, and reduces cytoskeletal integrity and focal adhesions with cells detaching even in static culture denoted by arrows. (*D* and *E*) Immunofluorescence of SQSTM/p62 (in green) and LAMP1 or HSP70 (in red) demonstrates an accumulation of SQSTM/p62 and LAMP1 positive vesicles, indicative of a block in autophagic flux. Detachment was observed under static conditions (arrows). (*F–K*) Changes in mRNA expression of key regulators of the chaperone-mediated autophagy pathway by OSGIN1 + 2 overexpression: (*F*) HSPA1A; (*G*) HSPA1B; (*H*) BAG3; (*I*) MAP1LC3B; (*J*) SQSTM1/p62; (*K*) ATG9A (mean ± SD, one-way ANOVA, **P < 0.01 and ***P < 0.001; *n* = 6).

Cell adhesion requires the integrity of the cytoskeleton and focal adhesions. Overexpression of OSGIN1&2 induced alterations in cell structure, with a collapse of the actin and tubulin networks (*Figure 6A–C*) and an overall reduction in staining for F-actin, tubulin, and vinculin (quantification Supplementary material online, *Figure S20*). Autophagic vesicles accumulated within HCAECs (*Figure**6D* and *E*) along with increased expression of genes involved in HSP70/BAG3-controlled chaperone-mediated autophagy pathway, confirming the RNAseq analysis (*Figure 6F–K*).

### 3.8 OSGIN1 and OSGIN2 regulate HCAEC adhesion

**Figure 7:**
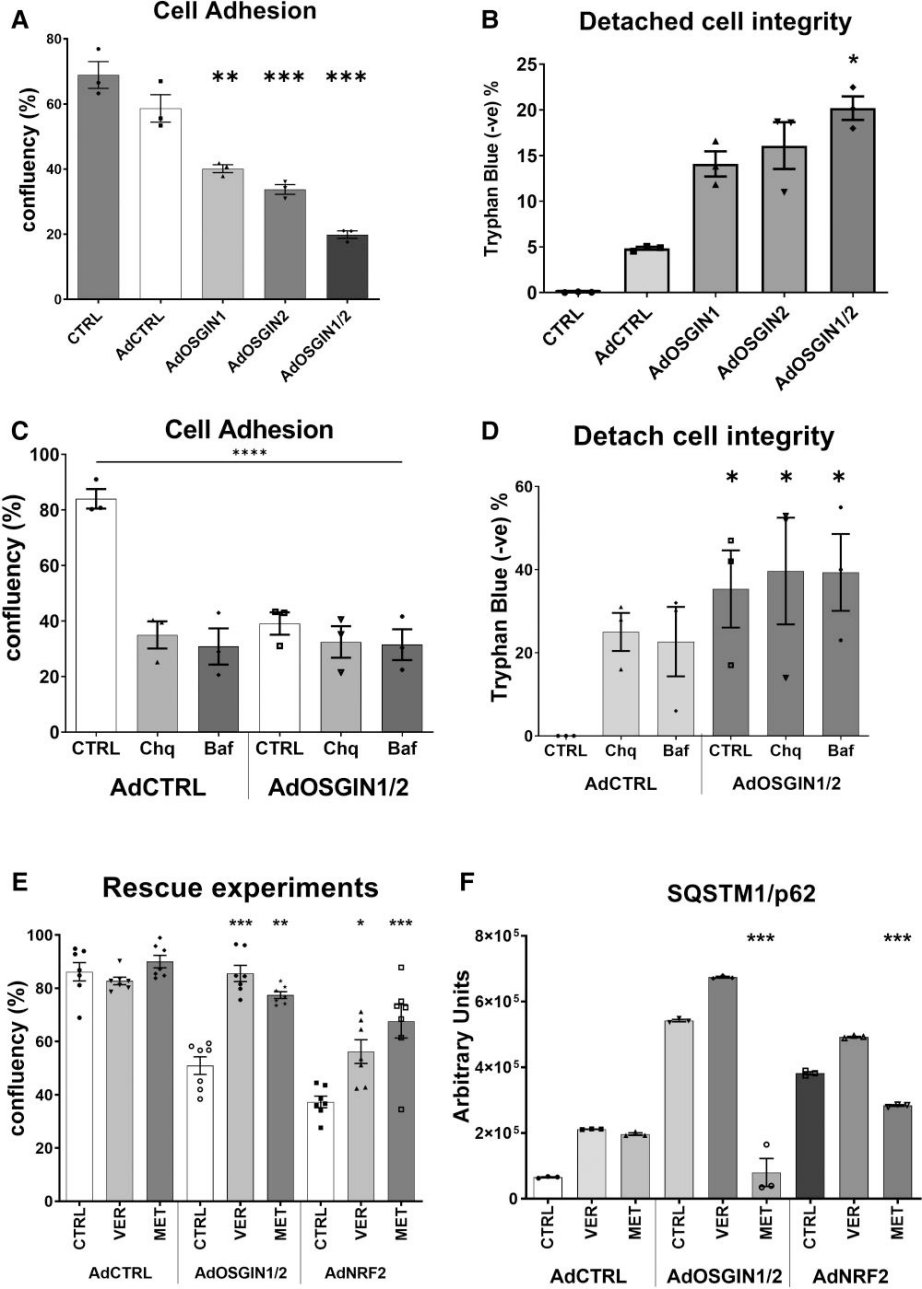
Quantification of endothelial cell detachment using orbital shaker model. (*A* and *B*) Adenoviral overexpression of OSGIN1, 2, and 1&2 triggers cell detachment (mean ± SD, one-way ANOVA, **P < 0.01, ***P < 0.001 vs. AdCTRL; *n* = 3), with detached cells displaying a significant maintenance of cell membrane integrity (*P < 0.05 vs. AdCTRL; *n* = 4). (*C* and *D*) Chloroquine (150 µM), bafilomycin (50 nM), or OSGIN1&2 overexpression induced comparable, non-synergistic detachment (mean ± SD, two-way ANOVA, **P < 0.01, ***P < 0.001 vs. AdCTRL, *n* = 4), with a similar maintenance of cell membrane integrity (*P < 0.05 vs. AdCTRL, *n* = 4). (*E*) Co-treatment with Ver155008 (15 µM) or Metformin (100 µM) reduced OSGIN1&2 or Nrf2-mediated cell detachment (mean ± SEM, two-way ANOVA, *P < 0.05, **P < 0.01, ***P < 0.001, *n* = 7). (*F*) Metformin, but not VER-155008 treatment reversed SQSTM1/p62 protein accumulation following AdOSGIN1 + 2 and AdNRF2 overexpression (mean ± SEM, two-way ANOVA, ***P < 0.001, *n* = 3).

Overexpression of OSGIN1&2 triggered HCAEC detachment, even in static culture (*Figure 6A*). We used the orbital shaker system to normalize the physical forces to which HCAECs were exposed between experiments. Overexpression of OSGIN1&2 (*Figure 7A–B*) yielded comparable levels of detachment to Nrf2 overexpression (*Figure 2A*). Of note, many of the cells that detached retained membrane integrity as assessed by Trypan Blue exclusion, suggesting detachment was not a consequence of a loss of cell viability. This clearly demonstrates that OSGIN1&2-regulated processes negatively regulate adhesion.

### 3.9 Inhibition of proteostasis regulates HCAEC adhesion

Given that OSGIN1&2 negatively regulates both adhesion and autophagy, we sought to understand if autophagy regulates adhesion in HCAECs. Incubation with relatively low doses of chloroquine (150 µM) or bafilomycin (50 nM) to reduce autophagic flux (*Figure**7C* and *D*), or moderate doses (300 and 100 nM; Supplementary material online, *Figure S17*), also triggered cell detachment with similar maintenance of membrane integrity, suggesting that normal autophagic flux is required for HCAEC adhesion. There was no additive effect of co-treatment of endothelial cells with chloroquine or bafilomycin and OSGIN1&2 overexpression suggesting that a reduction in autophagic flux by either chloroquine or bafilomycin, or by OSGIN1&2 overexpression may affect cell adhesion through the same mechanism.

### 3.10 HSP70 inhibition or activation of AMPK with metformin limits detachment

As OSGIN1&2 overexpression markedly enhanced the expression of HSP70 & BAG3 that are required for chaperone-mediated autophagy, we investigated if dysregulation of chaperone-mediated autophagy increases HCAEC detachment by inhibition of the HSP70 nucleotide binding site using VER-155008. HSP70 inhibition reduced both OSGIN1&2 and Nrf2-mediated HCAEC detachment (*Figure 7E*). Similarly, promotion of macroautophagy via 5′ AMP-activated protein kinase (AMPK) activation with metformin mitigated cell detachment following OSGIN1&2 or Nrf2 overexpression and reversed the accumulation of autophagic vesicle markers p62 and LAMP1 (*Figure**7E* and *F*; Supplementary material online, *FiguresS18* and *S19*). We also investigated if patients experiencing plaque rupture or plaque erosion have altered blood HSP70 concentrations. No overall difference was seen between patients with plaque rupture or plaque erosion (*n* = 46 rupture, 21.2 ± 10.2 vs. *n* = 34 erosion, 22.8 ± 9.0 pg/mL, *P* = 0.242; Supplementary material online, *Figure S20A*); however, when comparing smokers with plaque rupture or plaque erosion, HSP70 was elevated in the plasma of patients with plaque erosion (Supplementary material online, *Figure S20B*, *n* = 21 rupture, 18.4 ± 9.7 vs. *n* = 20 erosion, 24.1 ± 8.7 pg/mL, *P* = 0.033), with no difference in non-smokers (see Supplementary material online, *Figure S20C*, *P* = 0.22) or overall effect of smoking (see Supplementary material online, *Figure S20D*, *P* = 0.25).

## 4. Discussion

Multiple studies have identified an increased frequency of plaque erosion in patients who smoke.^4,6,9,13,14^ The data presented here identify a novel smoking-activated, Nrf2-driven mechanism that negatively affects HCAEC adhesion, which is amplified under elevated flow. The haemodynamic environment profoundly influences endothelial behaviour.^23–25^ Most clinically relevant plaque erosions occur where the endothelium is exposed to elevated flow,^29–32,43^ suggesting that the phenotype adopted by endothelial cells under elevated flow may be permissive or amplify pathological responses involved in erosion. This is the first study to provide a shear-regulated molecular pathway that might explain the association between smoking and elevated flow with plaque erosion.

Cigarette smoke contains high levels of free radicals, reactive oxygen, and nitrogen species.^44^ We and others have shown that aqueous CSE activates Nrf2-dependent gene expression in human endothelial cells.^21,22,38^ Physiological laminar flow modestly increases Nrf2 activity in the endothelium, contributing to athero-protective signalling.^45–47^ However, Nrf2 has other actions *in vivo* including modulation of lipid metabolism,^48^ increasing foam cell formation,^49^ and NLRP3 inflammasome co-activation.^50^ As a result, global deletion of Nrf2 in hypercholesterolemic mice reduces rather than increases atherosclerosis.^18^ These and our present observations raise a cautionary note with respect to chronic modulation of Nrf2 as an adjunctive therapy to prolong lifespan.^51^ Both sulforaphane and isoliquiritigenin are phytochemicals, highlighting that Nrf2 activity can be modulated by numerous pathways, including diet, smoking, and also exposure to airborne pollution.^52^

Cigarette smoke can induce senescence in a number of cell types.^53,54^ Overexpression of OSGIN1&2 reduced proliferation of HCAECs (*Figure**4C* and *D*) and induced markers of senescence (*Figure 4E–G*), and may, therefore, mediate smoking or oxidative stress-induced senescence^53,55^ and reduce tissue repair.^56^ The role of Nrf2 and OSGIN1&2 in regulating proliferation is intriguing, potentially performing a protective function that limits proliferation under oxidative stress prior to effecting (DNA) repair, inducing senescence if oxidative stress is sustained.

Smoking causes lung inflammation resulting in the release of inflammatory cytokines including TNFα into the circulation to an average of 30 ng/mL.^15^ Our data suggest that a flow-dependent synergy between oxidative stress and inflammatory cytokines elicits endothelial detachment, suggesting a two-hit mechanism of oxidative stress and inflammation that may be required to trigger erosion.

OSGIN1&2 increased the expression of genes related to proteostasis, including HSP70 and BAG3, and promoted the accumulation of p62-labelled vesicles, indicative of a block in autophagic flux. Consistent with this observation, treatment with chloroquine or bafilomycin, which inhibit autophagic flux, also triggered HCAEC detachment. The defect in adhesion could be partially restored by inhibition of HSP70, or activation of AMPK, which promotes macroautophagy. These observations support the hypothesis that functional autophagy is required for cell adhesion^57,58^ and that high-level activation of Nrf2 and OSGIN1&2 dysregulate chaperone-mediated autophagy, promoting cell detachment. Accumulation of p62 enhances Nrf2 signalling,^59,60^ thus inhibition of autophagic flux might create a positive feedback loop further increasing Nrf2 activation. Inhibition of HSP70, or activation of macroautophagy, may represent novel therapeutic strategies to limit oxidative stress-induced cell detachment.

Our transcriptomic data suggested that TLR and/or interferon signalling may synergize or amplify the gene expression pattern elicited by OSGIN1&2 overexpression because numerous interferon-regulatory factor (IRF) IRF3/7 binding sites in the promoters of OSGIN1&2 regulated genes. Although not studied here, the sub-endothelial matrix likely contributes to the maintenance of endothelial integrity in humans. For example, eroded plaques contain abundant hyaluronan and versican,^61^ and plaque erosion patients have increased expression of HYAL2 (which processes hyaluronan to proinflammatory lower molecular weight species) and the hyaluronan receptor CD44v6.^12^ Hyaluronan can bind to and activate TLR2 and TLR4.^62^ Engagement of TLR2 (potentially by hyaluronan fragments) stimulated endothelial apoptosis and detachment, which was enhanced by neutrophil NET formation.^3,63–65^ TLR-regulated gene expression via IRF3/7 may overlap with OSGIN1&2-dependent gene expression and synergize to affect endothelial function. We noted a 50-150-fold increase in the expression of HSP70 (HSPA1A&B; *Figure 5*). Extracellular HSP70 can also bind and activate TLR4 in ECs^66^ through TRIF-dependent signalling,^67^ potentially instigating a positive feedback loop through TLR4 that could promote cell detachment under these conditions.

The combination of CSE and TNFα elevated the expression of GDF15 in HCAECs, which we demonstrated was elevated in the circulation of patients who experience plaque erosion, with the effect predominating in patients who smoke. Similarly, HSP70 expression was elevated in smokers who experienced plaque erosion. Consistent with the published demographic differences between patients who experience plaque rupture and erosion, the patients in the plaque erosion group were significantly younger than those in the rupture group (51.1 ± 8.1 vs 59.7 ± 10.9), and we cannot exclude that the differences we observed in GDF15 and HSP70 levels are related to age; however, circulating GDF15 concentrations increases with age^68^ suggesting that the observed increase in GDF15 with erosion is likely robust. Lastly, we demonstrated that OSGIN1 and OSGIN2 were up-regulated in the aortas of mice exposed to cigarette smoke. While these observations provide corroborating evidence that the pathways identified here have clinical relevance, it is not possible to unequivocally demonstrate this in humans, as the cells required for the study have detached from the plaque and are, therefore, not available for the study to determine if the pathways identified here actively promote plaque erosion.

In conclusion, we created an *in vitro* model that combined the effects of erosion-prone flow with the insults derived from smoking to provide molecular insights into HCAEC dysfunction and adhesion. In an elevated flow environment, CSE and TNFα strongly up-regulated Nrf2 and led to endothelial detachment that was not mediated by apoptosis. Furthermore, overexpression of Nrf2 or downstream genes OSGIN1 and OSGIN2 caused endothelial detachment associated with dysregulated HSP70/BAG3-mediated proteostasis. The existence of two positive feedback loops with elevated p62 increasing Nrf2 activation and extracellular HSP70 increasing TLR2/4 activity may propagate pathological effects of smoking on HCAEC erosion. Taken together, our findings highlight a novel Nrf2-OSGIN1&2-proteostasis axis that regulates endothelial adhesion with potential relevance for ACS caused by plaque erosion and identify new targets for further investigation to explore pharmacological intervention.

## Supplementary material

Supplementary material is available at *Cardiovascular Research* online.

## Authors’ contributions

S.J.W., A.C.N., and Y.A. conceived and designed the research; S.S., R.B., X.L., A.L.-S., G.R.F., J.T., G.N., T.T.A., J.S., G.G.R., H.J., H.D., and G.H. acquired the data and provided key resources for analysis; S.S., R.B., X.L., and A.L.-S. performed statistical analysis; S.J.W., A.C.N., and P.L. drafted the manuscript; S.J.W., A.C.N., P.L., A.K., Y.A., M.J.H., G.G.R., H.D., T.W.J., H.J., B.Y., and J.L.J. made critical revision of the manuscript for key intellectual content.

## Supplementary Material

cvad022_Supplementary_Data

## Notes

Translational perspective Plaque erosion is responsible for 25–30% of acute coronary syndromes and most frequently occurs on stenotic plaques where the endothelium is exposed to elevated flow. Plaque erosion is more commonly observed in smokers and younger patients, particularly women. Herein, we provide a novel pathway that links the smoking-induced upregulation of Nrf2 with endothelial detachment and identify OSGIN1&2 as key players in this process. In addition, we show that detachment can be abrogated by AMPK activation or HSP70 inhibition, and that GDF15 and HSP70, identified by our model, are elevated in patients that experience plaque erosion compared to those that experience plaque rupture.
