# Supplementary material for: A Nrf2-OSGIN1&2-HSP70 axis mediates cigarette smoke-induced endothelial detachment: implications for plaque erosion
Source: Cardiovasc Res. 2023 Feb 18;119(9):1869–82. doi: 10.1093/cvr/cvad022 (PMC10405570; doi:10.1093/cvr/cvad022)
Supplement: cvad022_Supplementary_Data [file cvad022_supplementary_data.pdf]

A pivotal role for Nrf2 in smoking induced endothelial detachment– implications for endothelial erosion of stenotic plaques

Authors: Sandro Satta[1], Robert Beal[1], Rhys Smith[1], Xing Luo[2], Glenn R Ferris[1], Alex Langford-Smith[1], Jack Teasdale[3], Tom Tanjeko Ajime[4], Jef Serré[4], Georgina Hazell[3], Graciela Sala Newby[3], Jason L Johnson[3], Svitlana Kurinna[5], Martin J Humphries[5], Ghislaine Gayan-Ramirez[4], Peter Libby[6], Hans Degens[1,8], Bo Yu[2], Thomas Johnson[7], Yvonne Alexander[1], Haibo Jia[2], Andrew C Newby[3], Stephen J White[1*]

Supplementary Information

Methods

Tissue culture:

Shear stress models - Confluent monolayers of HCAEC were initially cultured for 24 hours under oscillatory (0±5 dynes/cm^2, ±0.5Pa, 1 Hz OSS), normal laminar (15 dynes/cm^2, 1.5Pa LSS), or elevated laminar shear stress (75 dynes/cm^2, 7.5Pa ESS) to allow cells to adapt to their shear environment using a parallel plate flow apparatus. Three treatments were administered, 16 hours apart (Figure 1A) of vehicle (control), TNFα (5ng/ml), CSE (10%), or both (T+C). HCAECs were cultured for a total of 72 hours, with 48 hours of treatment.
In addition, the orbital shaker model was also used [1], as this allows analysis of cells that have detached. For this system, cells were seeded on day 1 into 6 well plates, transduced with virus day 2 at a total pfu/cell of 400. Virus was removed on day 3 and the cells exposed to shear stress for 48 hours prior to analysis. 3 ml of MV2 fresh media was added and the plates were placed on an orbital shaker at 210 rpm for 48 hours to generate a defined shear stress pattern across the well, with low shear stress of approximately 4 dyn/cm^2 in the centre and high shear stress of approximately 13 dyn/cm^2 in the periphery. Compounds aimed at modulating cell adhesion (Table S1) were added to the HCAECs on commencement of exposure to shear stress.

Compounds	Work concentration	Brand	Dissolved in
Chloroquine	150 or 300μM	Sigma	MV2 media
Bafilomycin	50nM or 100nM	Sigma	DMSO
Metformin	100μM	Sigma	MV2 media
VER-155008	15μM	Sigma	DMSO

Table S1 Chemical compounds added during orbital shaker experiment

ELISA

The level of GDF15, HSP70, S100P and IL-8 in patients' serum with OCT-defined plaque rupture or OCT-defined plaque erosion was measured by ELISA kit according to the manufacturer's instructions (CUSABIO, China).

Construction of Adenoviral vectors

OSGIN1 and OSGIN2 were cloned into pCpG-free MCS (Invivogen). The coding regions were amplified by PCR using primers (OSGIN1: F-CCAAGATCTCCACCCACCATGAGCTCCTCCAGAAAGGAC; R-TGGGCTAGCGTTAGGGTGGCTTCCTGGTCTCCTTCC. OSGIN2: F-GTGAGATCTAGAGGCGAAAAGTTAACACCATGCCATTAGTTGAAGAAACTTC; R-GTAAGCTAGCTTTAAGCTATCCCATCTCCTCC) using KOD proofreading DNA-polymerase, introducing a 5' BglII site and 3' NheI site. Once inserted into the respective sites in pC-G-free MCS, the whole expression

cassette was shuttled into pDC511 (Microbix Biosystems, Canada) for adenoviral vector production as previously described [2].

Construction of lentiviral vectors
Overexpression of KEAP1 was facilitated using lenti-KEAP1. KEAP1 was amplified by PCR using KOD proofreading polymerase and primers SW787F 5' – GCGGATCCTCTAGAACACCATGCAGCCAGATCCC which created BamHI and XbaI sites, and SW788R 5' CCCAGTCGACAAGCTAGCCTCAACAGGTACAGTTCTGCTG, which created NheI and SalI sites post stop codon. This fragment was TA cloned into pGEMTeasy and shuttled into lentiviral overexpression vector 326 under the control of the CMV promoter, replacing GFP (326 expressing GFP was used as a control). shRNAs for OSGIN1 & 2 were designed using splash RNA (http://splashrna.mskcc.org/)[3] and the top 3 designs were used to replace the first 3 miRs in the miR17 cluster with appropriate mismatches to ensure incorporation of target strand into RISC as described by [4]. Constructs were synthesised by Eurofins and cloned into plentilox3.1 under the control of the U6 promoter. An empty vector (no-shRNA) was used as a control.

Cytotoxicity, viability and apoptosis analysis
Apotox-Glo triplex assay (Promega, Madison, USA) was performed in the HCAECs, according to manufacturer's instructions, and 40 hours after AdOSGIN1 and AdOSGIN2 infection. To this end, cells were plated into 96-well plates at a density of 5 x 10^4 cells per well. Results were normalized to DMSO treated control cells. Experiments were performed five times with eight replicates per condition. In addition, Caspase-3 activity was measured using a caspase-3 activity assay kit (Promokine kit), fluorescence was measured at λ max = 505 nm after 3 h of incubation with the substrate and normalized by the protein content of each sample. Each treatment had five technical replicates and performed in triplicate.

Cell cycle analysis in HCAECs
Cell cycle analysis was performed by flow cytometry on ethanol-fixed cells stained with 25 µg/ml propidium iodide (PI). Analyses were performed using a FACScan flow cytometer (FACS Calibur, BD Transduction Laboratories) using Modfit software. BrdU analysis was performed as described[2].

Western blotting of HCAEC lysates
HCAECs were lysed in SDS lysis buffer [2% SDS; 50 mM Tris pH 6.8; 10% glycerol]. Protein concentration in the lysate was quantified using bicinchoninic acid-assay (BCA, Pierce BCA Protein Assay). Between 12 and 20 µg of protein were loaded per lane on denaturing SDS–polyacrylamide gels and blotted onto Nitrocellulose or PVDF membranes. See Table S2 for further information. All experiments were conducted in triplicate.

	Primary ab	Blocking Solution	Secondary ab
OSGIN1 (Biorbyt orb100666)	Rabbit-antiOSGIN1 (1:1000) O/N 4°C	1% BSA	Monoclonal Secondary HRP-anti rabbit (1:1000-1h) in TBSTween
OSGIN2 (Biorbyt orb185683)	Rabbit-antiOSGIN2 (1:500) O/N 4°C	1% BSA	Monoclonal Secondary HRP-anti rabbit (1:1000-1h) in TBSTween
PARP cleavage (Cell Signaling D64E10)	Rabbit anti-PARP (1:1000) O/N 4°C	0,5% BSA	Monoclonal Secondary HRP-anti rabbit (1:2000-1h) in TBSTween
SQSTM1/p62 (Abcam ab56416)	Mouse anti-p62 (1:1000) O/N 4°C	3% Milk	Monoclonal Secondary HRP-anti mouse (1:5000-1h) in TBSTween
HSP70 (Abcam ab45133)	Rabbit anti-HSP70 (1:1000) O/N 4°C	5% Milk	Monoclonal Secondary HRP-anti rabbit (1:5000-1h) in TBSTween
LAMP1 (Abcam ab24170)	Rabbit anti-LAMP1 (1:1000) O/N 4°C	1,5% Milk	Polyclonal Secondary HRP-anti rabbit (1:500-1h) + Monoclonal Secondary HRP-anti rabbit (1:500-1h) in TBSTween

Table S2. Primary Antibodies, dilutions and methodology used in Western Blotting

Immunocytochemistry of HCAEC

HCAECs were exposed to AdOSGIN1 and AdOSGIN2 infection before being fixed in cold 4% paraformaldehyde for 10 minutes. Cells were permeabilised with 0.1% triton, blocked in 20% goat serum and probed with rabbit anti-OSGIN1 (1:100, Biorbyt) and rabbit anti-OSGIN2 (1:75, Biorbyt) , and rabbit anti-VE-Cadherin (Cell Signaling) followed by goat anti rabbit Alexa Fluor 488 (1/200, Invitrogen). Further staining was carried out with mouse anti-Vinculin (1/400, Sigma), mouse anti-β-catenin (1/200, BD Transduction Laboratories), mouse -mab137 (1/100, Sigma) followed by goat anti mouse Alexa Fluor 594 (1/200, Invitrogen). In combination with the previous staining, anti-Phalloidin (1/250, Sigma), and anti-Tubulin (1/1000, Abcam) were added (antibody titration Table S3).

Antibody	Type	Dilution	Company
OSGIN1	Rabbit	1:100	(Biorbyt orb100666)
OSGIN2	Rabbit	1:75	(Biorbyt orb185683)
VE-Cadherin	Rabbit	1:400	(Cell Signalling D87F2)
β-Catenin	Mouse	1:100	(BD Transduction Laboratories 610153)
Vinculin	Mouse	1:400	(Sigma V4505)
Mab113	Mouse	1:100	(Abcam ab92824)
Tubulin	Already conjugated (Green)	1:1000	(Abcam ab64503)
Phalloidin	Already conjugated (Red)	1:250	(Sigma P1951)
HSP70	Rabbit	1:400	(Abcam ab45133)
LAMP1	Rabbit	1:100	(Abcam ab24170)
SQSTM1/p62	mouse	1:200	(Abcam ab56416)

Secondary Antibody description

Alexa fluor488	Anti mouse or rabbit	1:200	Invitrogen
Alexa fluor647	Anti mouse or rabbit	1:200	Invitrogen

Table S3. Primary Antibodies used in Immunocytochemical analysis

Senescent-associated β-galactosidase staining

Paraformaldehyde fixed HCAECs were rinsed with ice-cold PBS pH 6.0 and submerged in senescent-associated β-galactosidase (SA-β-Gal) staining solution [1 mg/ml 5-bromo-4-chloro-3-indolyl β-d-galactopyranoside (X-gal), 5 mM $K_4Fe(CN)_6$, 5 mM $K_3Fe(CN)_6$, 150 mM $NaCl_2$ and 2 mM $MgCl_2$ titrated with 1M NaH_2PO_4 to pH 6.0] and incubated overnight at 37°C. After staining, HCAECs were rinsed with ice-cold PBS pH 6.0, and micrographs were taken with a Leica M165 FC stereomicroscope.

RNA extraction and reverse transcriptase-polymerase chain reaction (RT-PCR)

Total RNA was extracted from HCAECs using the RNeasy Mini Kit (Norgen) according to the manufacturer's protocol. 250ng RNA were reverse transcribed into cDNA with random primer by reverse transcriptase (RT) (Qiagen). cDNA (relative 1ng RNA) was amplified by standard PCR with Taq DNA polymerase (Sensifast, SybrGreen, LOW-ROX Kit, Bioline) and primers. Primers were designed using NCBI software. For each gene, SYBR Green was used in place of a labelled probe (primers sequences Table S4). GAPDH RNA was used as the

internal control for each gene and the target gene was amplified in duplex in PCR mixtures (10 µl final volume) containing 4 µl Sybr® Green PCR Master Mix, cDNA template 1 µl, optimised primers 2 µl and 3 µl of H_2O. PCR thermal cycle parameters were: 5 minutes 95°C 30 seconds between at 65°C to 70°C depending on primers optimisation, 30 seconds at 72°C 40 cycles of 95°C for 15 seconds and 60°C for 1 minute. Reactions were performed, and fluorescence was monitored in an Applied Biosystem detector (Applied Biosystem). Relative mRNA expression level was defined as the ratio of target gene expression level to GAPDH mRNA expression. Primers sequences can be found in Table S4.

p21 (SW878F/879R)	F CTCAGGGTCGAAAACGGCGG<br>R GTGGGCGGATTAGGGCTTCCT
p16 (SW926F/927R)	F CGAGCTCGGCCCTGGAG<br>R TCGGGCGCTGCCCATCAT
HSPA1A (SW355F/356R)	F TGAGGAGCTGCTGCGACAGT<br>R GGCTGGAAACGGAACACTGG
HSPA1B (SW357F/358R)	F TGTTGAGTTTCCGGCGTTCC<br>R AACACCCCCACGCAGGAGTA
BAG3 (SW966F/967R)	F GCGGGGCATGCCAGAAACCA<br>R CTGGCCGGGTAACGTTCTGCT
ATG7 (SW894F/895R)	F GGACTGGCCGTGATTGCAGGA<br>R ATCCGATCGTCACTGCTGCTGG
ATG9A (SW938F/939R)	F AGAGGCGCTACGGTGGCATC<br>R GCCTTGATGCCGACTGCCCA
MAP1LC3B (SW934F/935R)	F CGCCCAGATCCCTGCACCAT<br>R AGCATTGAGCTGTAAGCGCCTTCT
SQSTM1/p62 (SW936F/937R)	F GGACGGGGACTTGGTTGCCTT<br>R CGGGTTCCTACCACAGGCCC
GABARAPL1/ATG8 (SW940F/941R)	F CGGACAGGGTCCCCGTGATTG<br>R AGCACTGGTGGGAGGGATGGT
GAPDH (SW180F/181R)	F CGGATTTGGTCGTATTGGGCG<br>R GCCTTCTCCATGGTGGTGAAGAC

Table S4. Primer sequences used for real-time PCR

RNASeq data analysis on HCAEC with OSGIN1&2 overexpression

Strand-specific RNA-seq libraries were prepared using the Illumina workflow with the TruSeq® Stranded mRNA Sample Preparation Kit. Paired-end reads of 65bp were generated from each sample on the Illumina platform of HiSeq4000. The fastq files generated were analysed with FastQC[5], any low quality reads and contaminated barcodes were trimmed with Trimmomactic[6]. All libraries were aligned to the hg38 assembly of human genome using STAR-2.5.3a[7] and only the unique alignments were reported for each read. The mapped reads were also counted with STAR at gene level against gencode.v25.annotation.gtf. R was used for all the statistical analysis of data[8]. The counts data were normalized and donor effect removed using the R package RUVSeq[9]. Differentially expressed genes were detected with the R package of DESeq2[10] between groups of experimental data sets. Cluster analysis was carried out on the DE genes identified with DESeq2 using a padj cut off of 0.05 with gplots[11].The predicted upstream regulators and altered canonical pathways were generated through the use of IPA (QIAGEN Inc., https://www.qiagenbioinformatics.com/products/ingenuity-pathway-analysis)[12]. The cluster analysis was carried out on the differentially expressed genes identified with DESeq2 using a p-adjusted cut off of 0.05, absolute log2 fold change cut off of 0.5, and base Mean cut off of 50. The Pearson distance was clustered with the hclust function and plotted with an R package of gplots.

Patient study population and blood samples

Patients presenting with ST-segment elevation myocardial infarction (STEMI) underwent primary percutaneous coronary intervention (PCI) at the Second Affiliated Hospital of Harbin Medical University were prospectively enrolled in this study. The diagnosis of STEMI was made according to the following criteria: detection of cardiac troponin values with at least one value above the upper reference, typical symptoms of acute myocardial ischemia and new ischemic electrocardiogram changes. Thrombus aspiration was performed in all patients before optical coherence tomography (OCT) examination. OCT imaging of culprit lesion were performed after antegrade flow restoration without any intervention. Patients were divided into plaque erosion and plaque rupture group according to OCT image of culprit lesion. The plaque rupture and plaque erosion were defined by OCT based on our previously established definition[13]. OCT-erosion was identified by the presence of attached thrombus overlying an intact and visualized plaque, while OCT-rupture was identified by disruption of fibrous cap and cavity formation in the plaque. The blood samples around the culprit lesion were collected from intracoronary aspirates during the primary PCI. The serum was separated and stored at -80°C immediately. Written informed consent was obtained from all patients. This study was approved by the institutional research ethics committee of the Second Affiliated Hospital of Harbin Medical University and conforms to the principles outlined in the Declaration of Helsinki.

	PR(n=46)	PE(n=34)	*p* value
**Demographic data**			
Female gender, n (%)	14(30.4)	7(20.6)	0.161
Age, year	59.7±10.9	51.1±8.1	<0.001
Smoking, n(%)	21(45.7)	20(58.8)	0.122
Hypertension, n(%)	17(36.9)	15(44.1)	0.259
Diabetes, n(%)	9(19.6)	4(11.8)	0.175
Hyperlipemia, n(%)	10(21.7)	8(23.5)	0.425
Stroke, n(%)	8(17.4)	4(12.1)	0.243
BMI (kg/m^2)	25.1±3.3	24.8±2.8	0.384
**Laboratory data**			
TC (mmol/L)	5.0±1.2	4.5±1.3	0.060
TG (mmol/L)	1.4(0.8-1.8)	1.2(1.0-1.9)	0.240
LDL (mmol/L)	3.2±1.0	2.9±0.8	0.112
HDL (mmol/L)	1.4±0.3	1.4±0.4	0.474
White blood cell (10^9/L)	10.8±3.6	12.2±4.1	0.061
C-reactive protein (mg/L)	4.0(1.6-7.7)	5.0(2.3-10.1)	0.218

Baseline characteristics of ACS patients analysed in this study that experienced either plaque rupture (PR) or plaque erosion (PE)

Immunofluorescence on Mouse Aortas

8µm frozen sections of aortas from mice exposed to air (control mice) or cigarette smoke for 3 months (as described[14]) were fixed in ice cold acetone before immunofluorescence was performed using the antibodies described above. The mouse tissue used in this manuscript was not generated for this study, but was surplus to the described study[14], and therefore utilised in this study in line with 3Rs principles. All experimental procedures were carried out in accordance with relevant guidelines and regulations and approved by the Ethical Committee of Animal Experiments of the KU Leuven.

SUPPLEMENTARY DATA

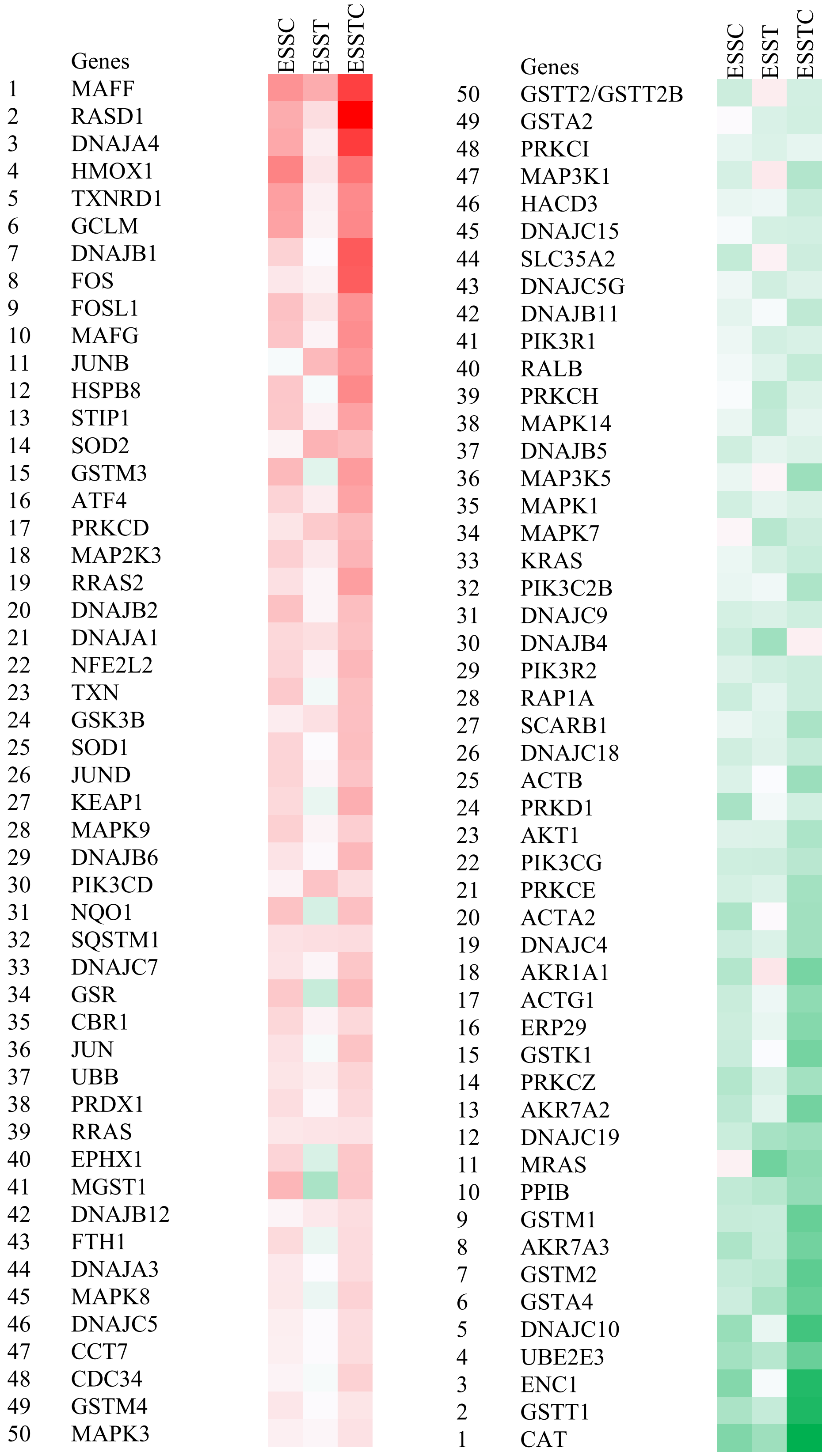

Figure S1. Top 50 genes upregulated and downregulated compared to elevated shear stress (ESS) control.

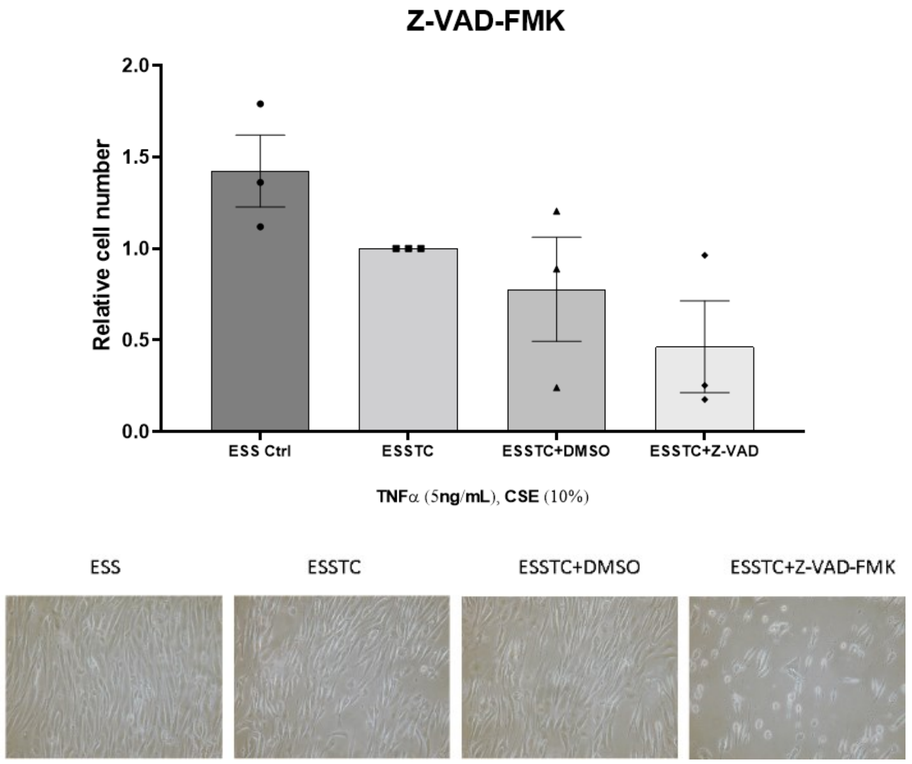

Figure S2: Cell Adhesion of HCAECs exposed to a combination of elevated shear stress, TNFα and CSE together (ESSTC), with additional Z-VAD-FMK treatment (20µM), vs untreated and DMSO controls at ESS for 72 hours. Cell number was quantified using picogreen assay, expressed as mean fold change against control ± S.E. n=3.

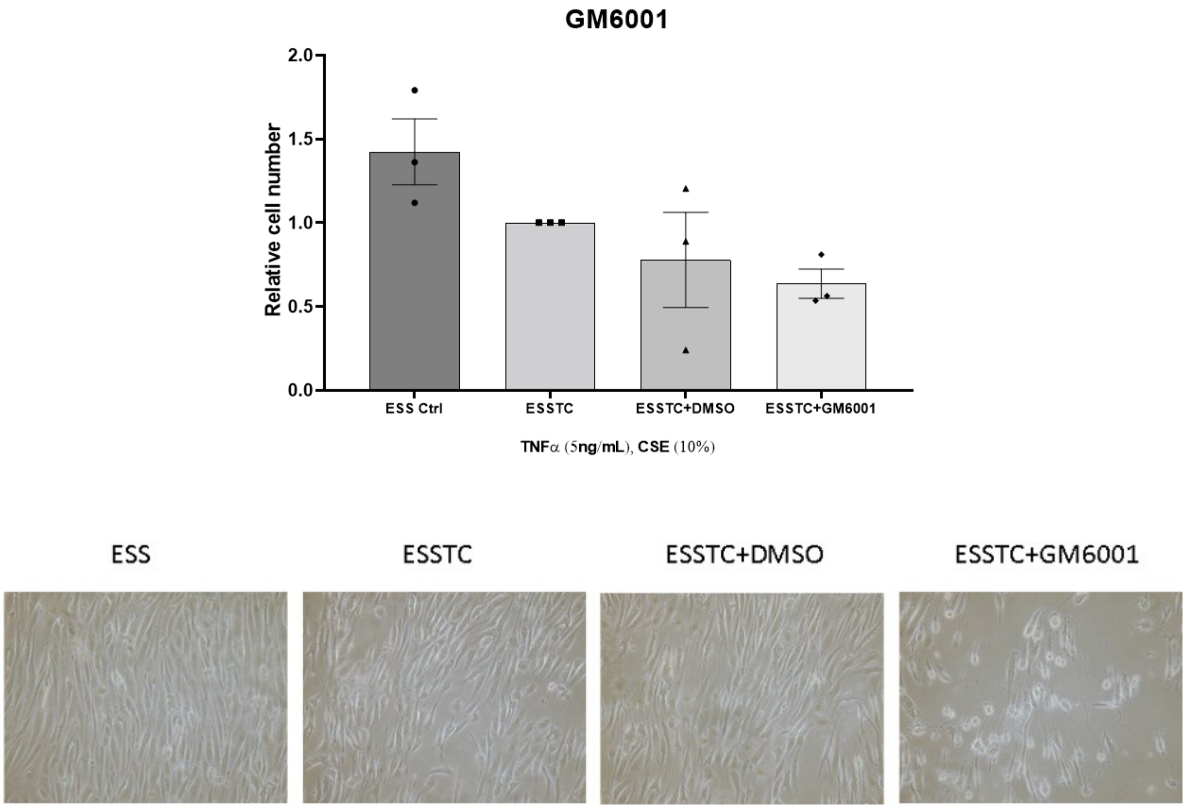

Figure S3: Cell number of HCAECs exposed to a combination of TNFα and CSE together (T+C), with additional GM6001 treatment (10µM), vs untreated and DMSO controls at ESS for 72 hours. Cell number was quantified using picogreen assay, expressed as mean fold change against control ± S.E. n=3.

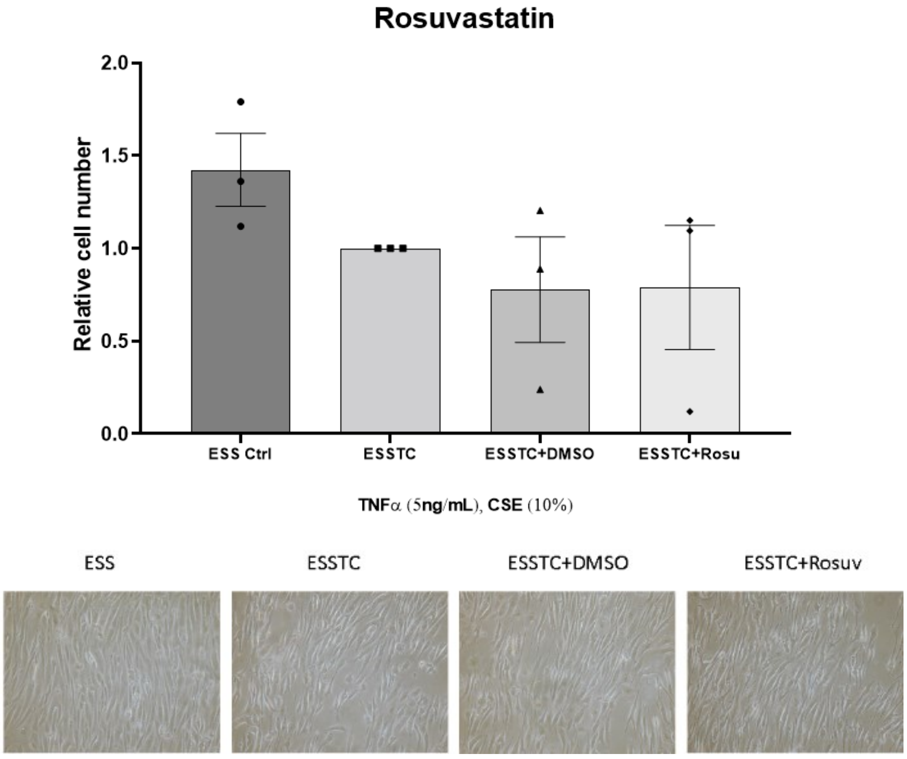

Figure S4: Cell Adhesion of HCAECs exposed to a combination of elevated shear stress, TNFα and CSE together (ESSTC), with additional 3µM Rosuvastatin treatment, vs untreated and DMSO controls at ESS for 72 hours. Cell number was quantified using picogreen assay, expressed as mean fold change against ESSTC ± S.E. n=3.

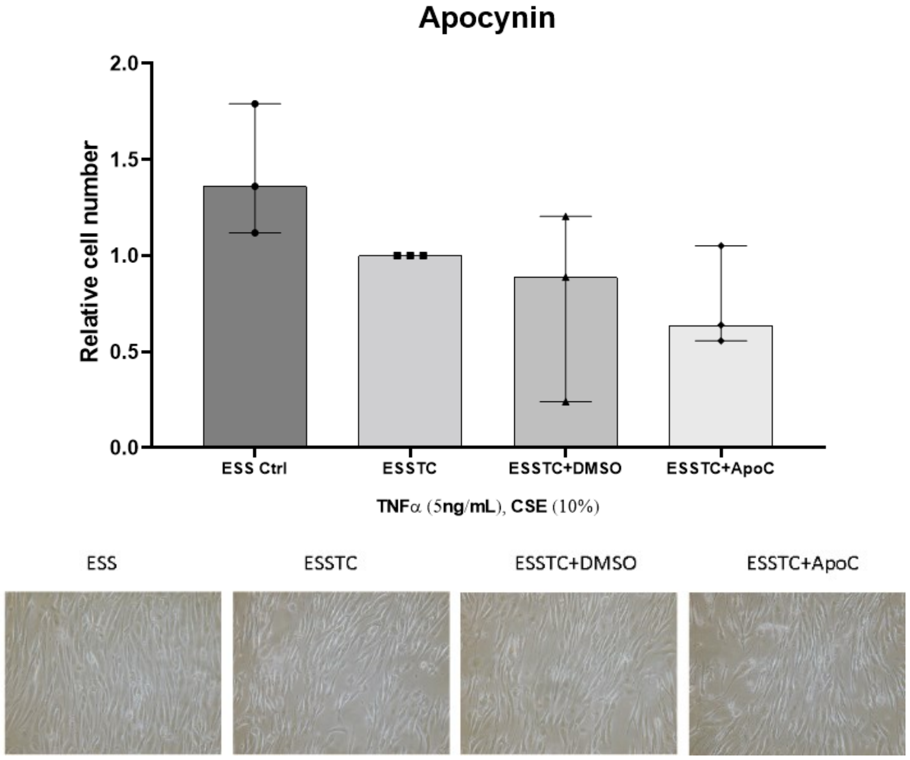

Figure S5: Cell Adhesion of HCAECs exposed to a combination of elevated shear stress, TNFα and CSE together (ESSTC), with additional 200µM Apocynin treatment, vs untreated and DMSO controls at ESS for 72 hours. Cell number was quantified using picogreen assay, expressed as mean fold change against control ± S.E. n=3.

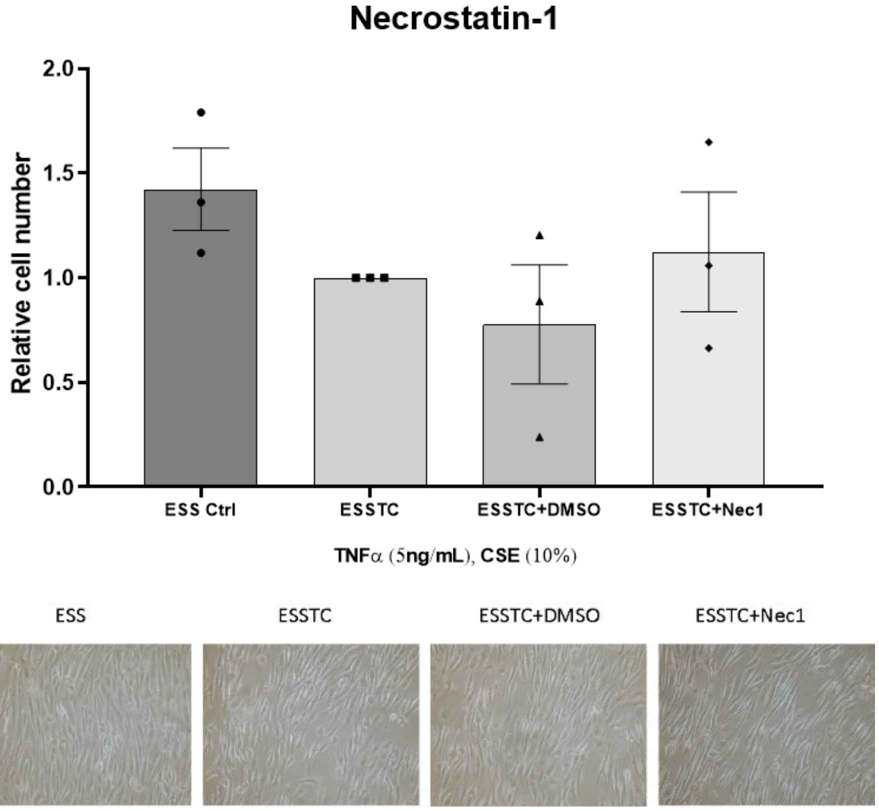

Figure S6: Cell Adhesion of HCAECs exposed to a combination of elevated shear stress, TNFα and CSE together (ESSTC), with additional Necrostatin-1 treatment (10µM), vs untreated and DMSO controls at ESS for 72 hours. Cell number was quantified using picogreen assay, expressed as mean fold change against control ± S.E. n=3.

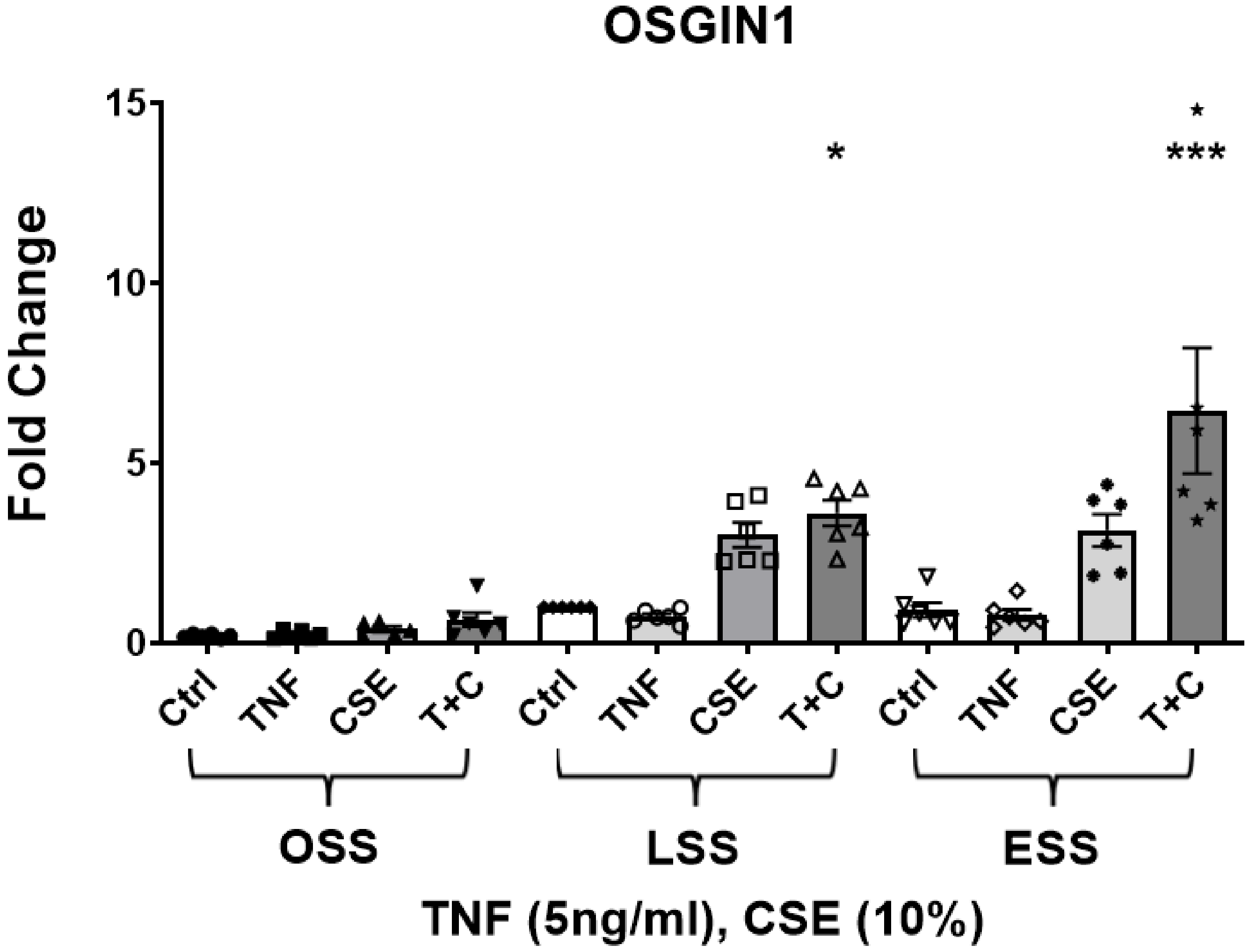

Figure S7. OSGIN1 mRNA expression in HCAECs cultured under OSS, LSS or ESS, with TNFα (5ng/ml) or CSE (10%) or both (*P=0.05, **P<0.01, ***P<0.001 v LSS CTRL, mean and SEM n=6, two-way ANOVA). Reproduced from reference [17], under CCL4.0

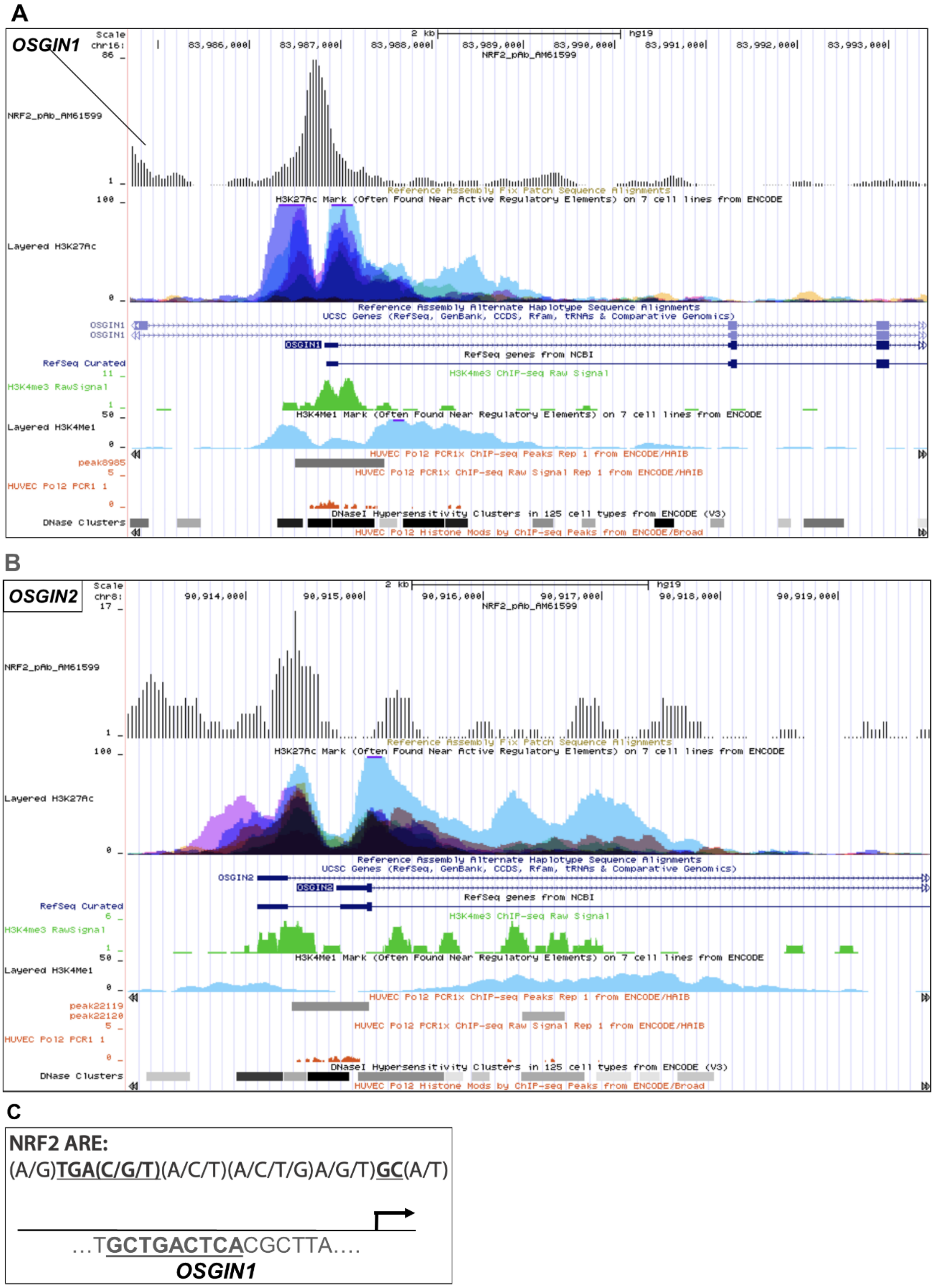

Figure S7. NRF2 binds and activates OSGIN1 promoter (A, B) UCSC genome browser tracks displaying ChIP-seq reads for NRF2 (grey), layered H3K27Ac (shades of blue), H3K4me3 (green), H3K4me1 (light blue), and POLII (grey bars and orange peaks). DNase hypersensitive clusters are in black. Please note higher scale of NRF2 and H3K4me3 at the promoter of OSGIN1, together with the higher levels of H3K4me1 on OSGIN1 compared to OSGIN2. While POLII is bound to promoters of both genes, difference in H3K4 methylation suggests that OSGIN1 is actively transcribed and OSGIN2 is poised for transcription. We found an NRF2 binding site antioxidant response elements (ARE) under the NRF2 binding peak located at 130bp upstream of the transcription start site (TSS) of OSGIN1 (C) and predicted four AREs 4kb upstream of the OSGIN2 TSS. This supports our previously published findings[15].

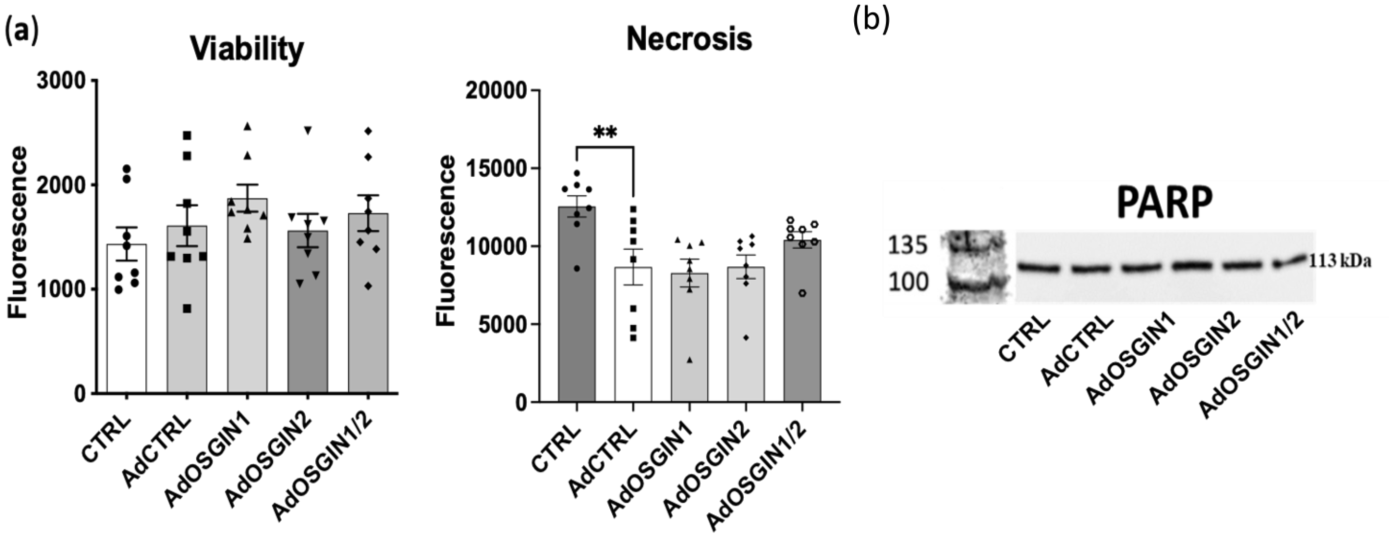

Figure S8 **a)** Cell viability assay for HCAECs. The cell viability was measured post transfection at 24h (adenovirus overexpression of OSGIN1, OSGIN2 and both together) and did not cause a significant decrease in cell viability. Overexpression of OSGIN1, OSGIN2 and both together did not cause a significant increase or decrease in necrosis. Viability and Necrosis were measured using promega Apotox Glo assay kit. **c)** Total Parp antibody in western blotting showed no parp cleavage by caspase-3. No second band was reported in any sample suggesting no apoptosis activity.

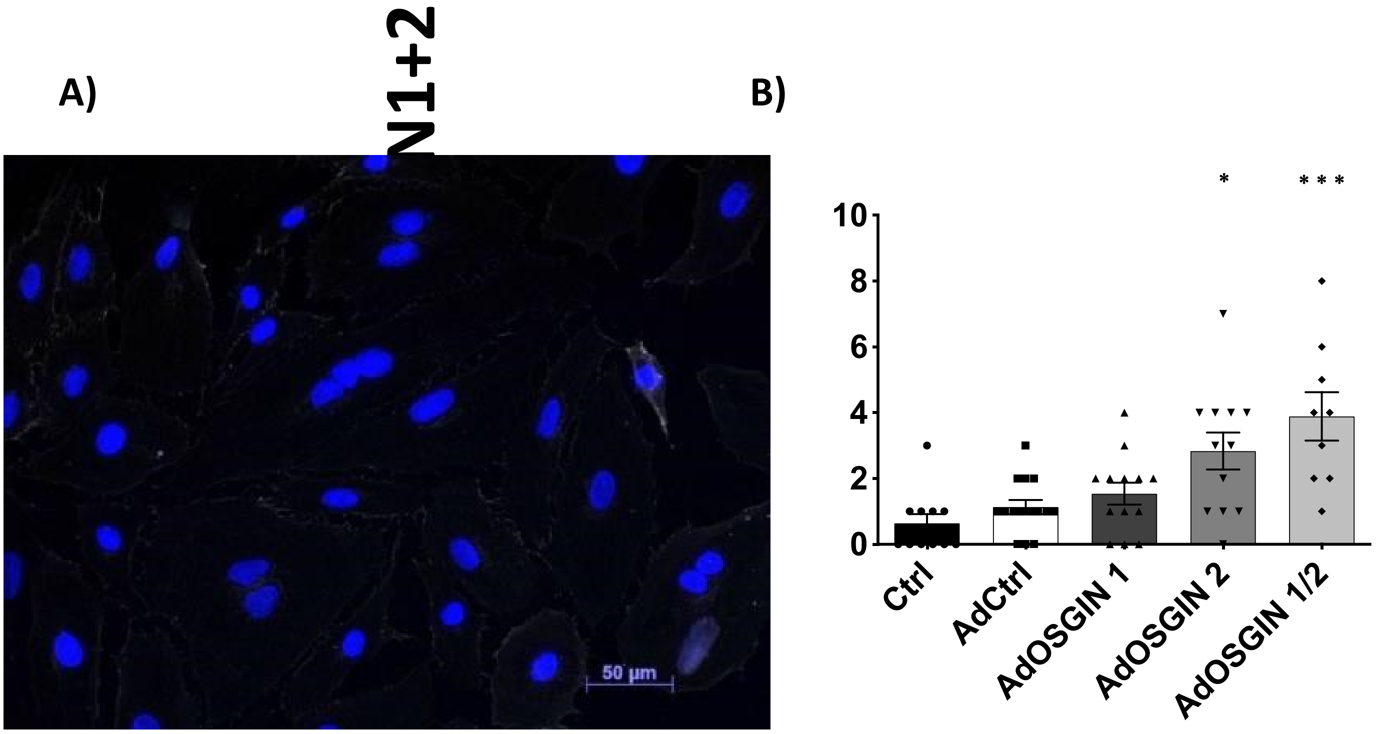

Figure S9. A) AdOSGIN1/2 condition resulted in numerous multinucleated cells. B) Number of multi-nucleated cells were counted and compared against AdCtrl. AdOSGIN2 and AdOSGIN1/2 showed up to four times more multi nucleated cells *P<0.05, and ***P<0.001.

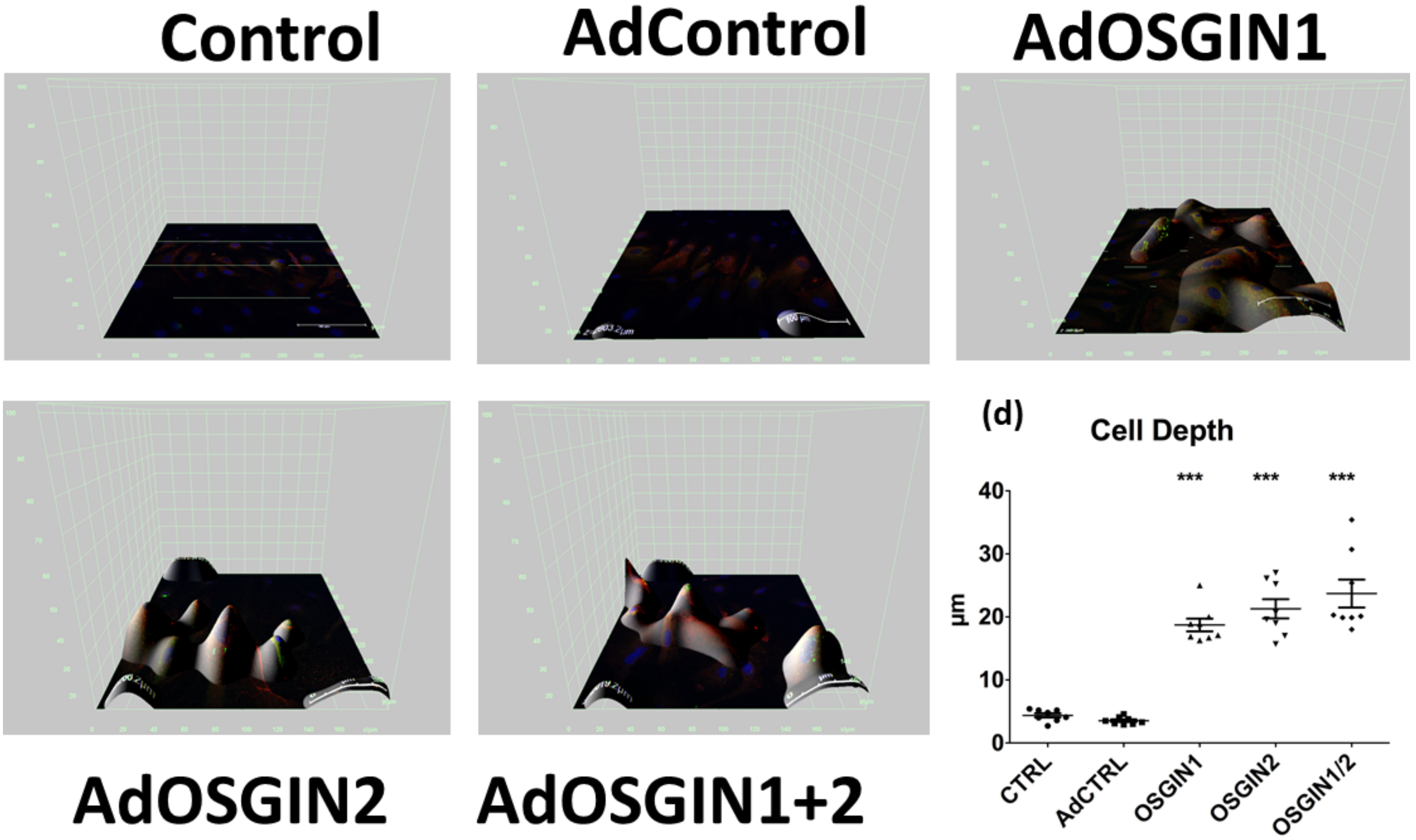

Figure S10: 3D cell model was created using Leica software confocal image stack in combination with Image j: image to stack from 50 layers of confocal images. Cell depth was measured with an increment of cell height up to 25µm (n=3; P<0.001).

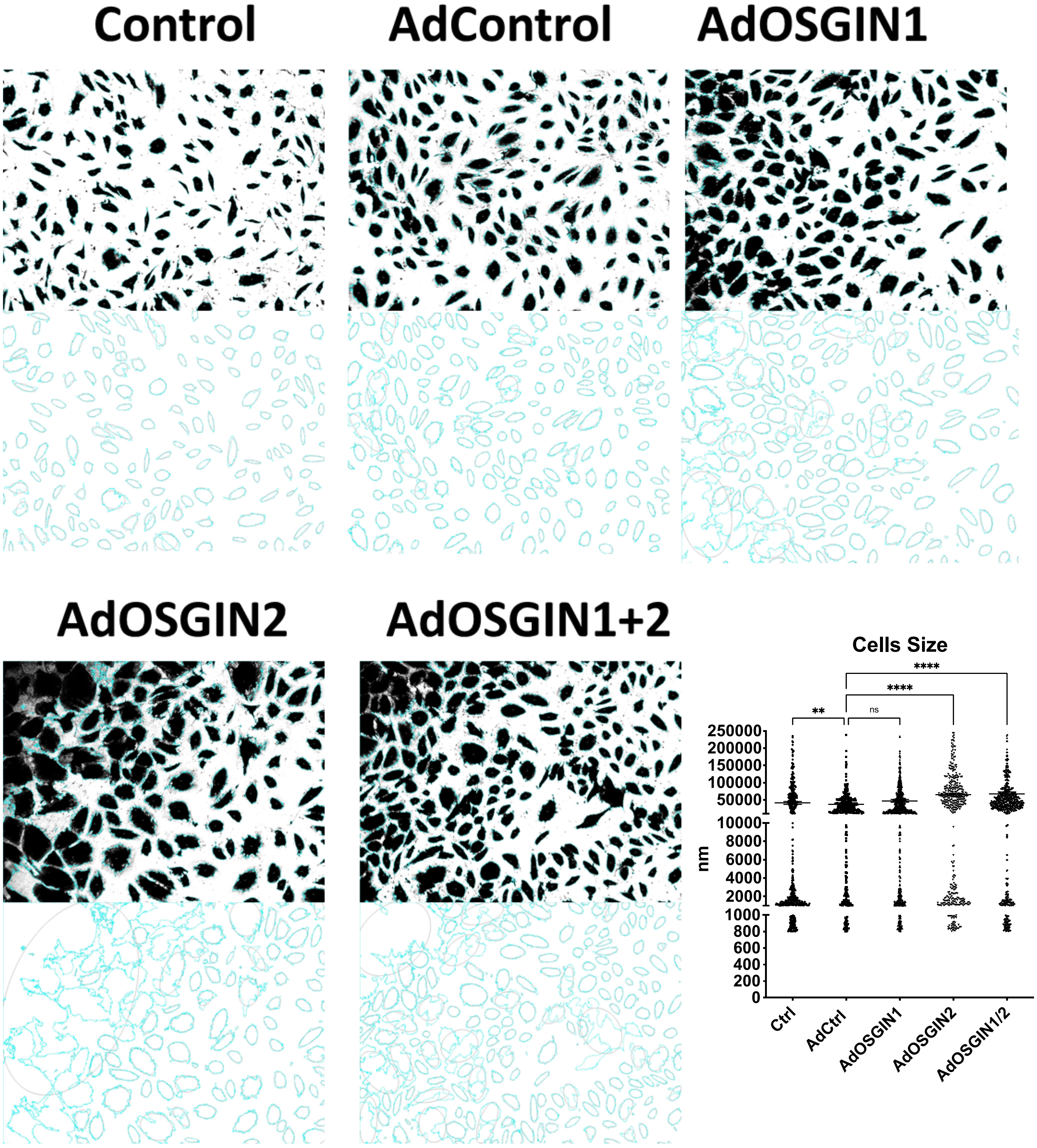

Figure S11: EC size analysis was evaluated through Image J custom particles analyser and multi cell outliner (ellipse fit correction). Five random pictures were analysed, and each cell area was automatically measured. AdOSGIN2 and AdOSGIN1+2 condition showed an increment in cell size with almost double the size compared to the Ctrl and AdCtrl. **P<0.01 AdOSGIN2 and ***P<0.001 AdOSGIN1+2 vs AdCtrl.

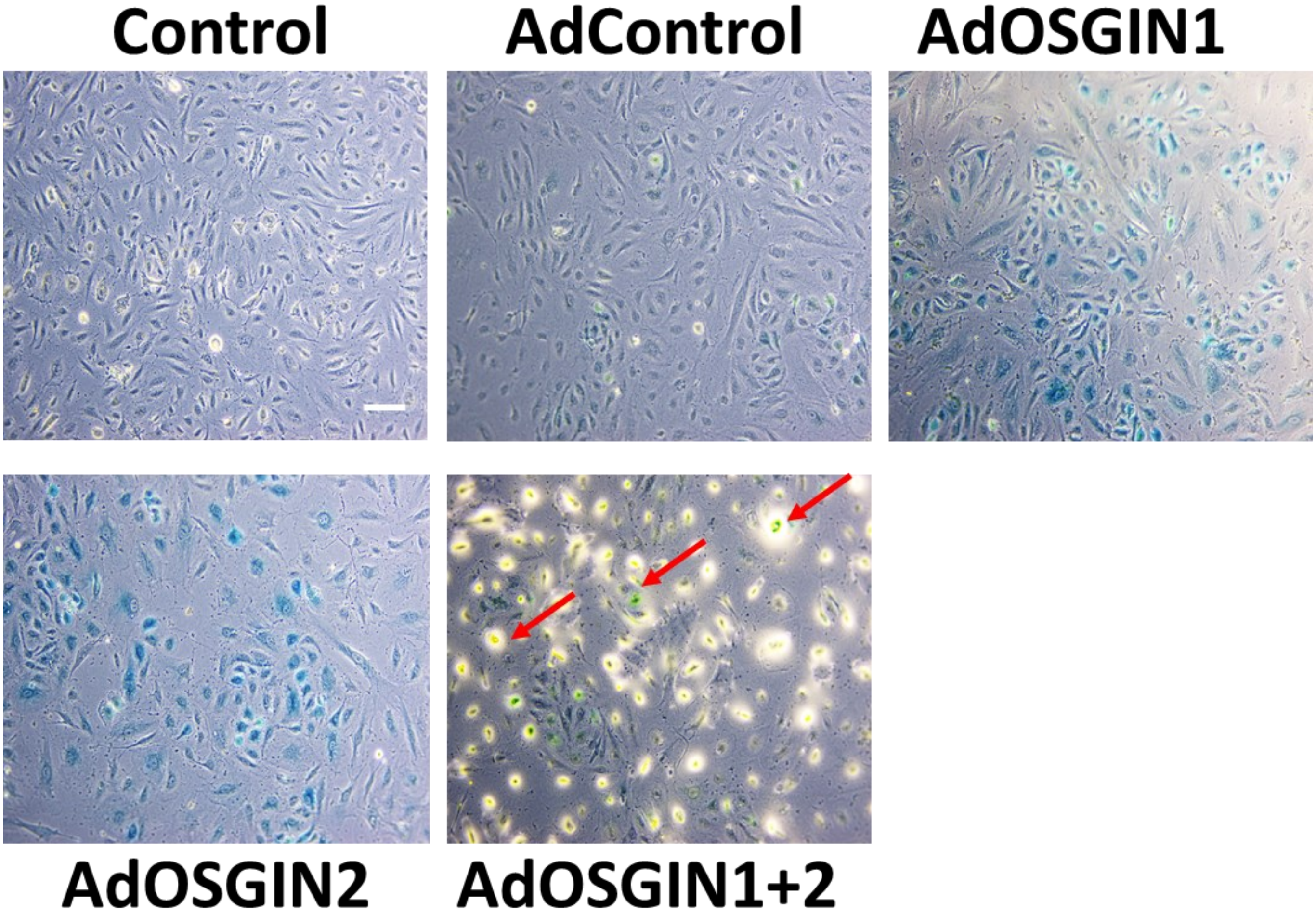

Figure S12. Senescence-associated beta-galactosidase (SA-β-galactosidase) senescence staining of HAECs transfected with adenoviral (OSGIN1, OSGIN2 and OSGIN1/2). Overexpression by ad of AdOSGIN1 and AdOSGIN2 induces senescent phenotypes in HCAECs. AdOSGIN1/2 overexpression shows green/blue senescence cells (red arrows) detached or in progress of detaching. SA-β-galactosidase assays were performed 40h after adenovirus transfection.

Supplementary Tables S5 Cluster 1, Genes and top 10 canonical pathways and regulators.

**Genes**	
AHSA1	HSPB1
ALDH3A2	HSPB8
ATF3	HSPD1
B3GAT3	HSPE1
BAG3	HSPH1
BANF1	IER5L
C3orf52	IL7R
CACYBP	JMJD6
CBARP	LGALS8
CHKA	LUC7L3
CHORDC1	MICB
CPNE8	MLKL
CRYAB	MRPL18
CRYZ	MSX1
DEDD2	NAPG
DNAJA1	NDRG1
DNAJB1	OSGIN1
DNAJB6	P4HA2
EVI2B	PATL1
FKBP4	PAXBP1
FTL	PLAUR
GDF15	PLEKHB2
HIF3A	PRPF38B
HIST2H2BE	PTGES3
HMOX1	RNF19B
HSP90AA1	RSRP1
HSP90AB1	SNX3
HSPA1A	SPATS2L
HSPA1B	SQSTM1
HSPA4	STIP1
HSPA6	TRIM16
HSPA8	TSPYL2

**Ingenuity Canonical Pathways**
Aldosterone Signaling in Epithelial Cells
Protein Ubiquitination Pathway
NRF2-mediated Oxidative Stress Response
Unfolded protein response
Glucocorticoid Receptor Signaling
eNOS Signaling
Aryl Hydrocarbon Receptor Signaling
Huntington's Disease Signaling
Xenobiotic Metabolism Signaling

**Upstream Transcriptional Regulator**
HSF1
FBXW7
PML
SP100
NFE2L2
TP53
ETS1
HSF2
HTT
NUPR1

Supplementary Tables S6 Cluster 2, Genes and top 10 canonical pathways and regulators.

Genes	
ANKRD10	MX1
APOL6	MX2
BATF2	OAS1
C19orf66	OAS2
DDX58	OASL
DDX60	OSGIN2
DDX60L	PARP14
DHX58	PARP9
DNAJA4	PLSCR1
DTX3L	PMAIP1
EIF2AK2	PPP1R18
EPSTI1	RSAD2
GBP1	SAMD9
GCH1	SAMD9L
HERC6	SERPINH1
IFI35	SLC15A3
IFI44L	SLC37A1
IFIH1	SP110
IFIT1	TAP1
IFIT2	TRIM21
IFIT3	TRIM26
IFIT5	TRIM69
IFITM1	UBC
IRF7	UBE2L6
ISG15	XAF1
LY6E	

Ingenuity Canonical Pathways
Interferon Signaling
Activation of IRF by Cytosolic Pattern Recognition Receptors
Role of Pattern Recognition Receptors in Recognition of Bacteria and Viruses
Role of RIG1-like Receptors in Antiviral Innate Immunity
Tetrahydrobiopterin Biosynthesis I
Tetrahydrobiopterin Biosynthesis II
Retinoic acid Mediated Apoptosis Signaling
Toll-like Receptor Signaling
Death Receptor Signaling
Protein Ubiquitination Pathway

Upstream Transcriptional Regulator
IRF7
STAT1
IRF1
NKX2-3
IRF3
IRF5
TRIM24
CNOT7
STAT3
STAT2

Supplementary Tables S7 Cluster 3, Genes and top 10 canonical pathways and regulators.

**Genes**	
ADM	PRDX6
ANGPT2	SBDSP1
F2RL2	SEC61G
GCLM	SELO
INA	SLC17A9
LENG9	SNRPA1
ME1	TBXAS1
MLLT11	TKT
NQO1	TMEM156
P4HA1	TRIM16L
PDE4B	UPP1
PLPP2	XPOT
PRDX1	ZDHHC6

**Ingenuity Canonical Pathways**
NRF2-mediated Oxidative Stress Response
Glutathione Biosynthesis
Pentose Phosphate Pathway (Non-oxidative Branch)
Superoxide Radicals Degradation
Prostanoid Biosynthesis
Phagosome Maturation
Pentose Phosphate Pathway
γ-glutamyl Cycle
Glutathione Redox Reactions I
Gluconeogenesis I

**Upstream Transcriptional Regulator**
MAFG
BACH1
NFE2L2
KEAP1
MAFK
NFE2
PML
KLF2
NFE2L1
PDX1

Supplementary Tables S8. Cluster 4, Genes and top 10 canonical pathways and regulators.

**Genes**
ANGPTL4
CTD-2015G9.2
HOXA9
HOXB7
MT-CYB
RPSAP58
TNFSF18

**Ingenuity Canonical Pathways**
Oxidative Phosphorylation
Mitochondrial Dysfunction
Sirtuin Signaling Pathway

**Upstream Transcriptional Regulator**
PHF1
COMMD3-BMI1
KMT2A
MLLT1
ASB2
NKX2-3
PSIP1
HOXB3
BHLHE41
PLAGL1

Supplementary Tables S9. Cluster 5, Genes and top 10 canonical pathways and regulators.

**Genes**	
ALDH1A3	MFSD11
AMDHD2	NUP107
ANAPC5	NUP160
ANKRD33B	PACS1
AP3M2	PAN2
ATXN2	PCMTD2
C22orf29	PIGQ
CAPN11	PLA2G4C
CHD1L	PLD1
CLIP4	PPCS
CPNE5	RCBTB1
DFNA5	RFC3
DHRS1	RNF123
DHRS11	SESTD1
EEF2K	SLC35E2B
EVL	SMARCC2
HAS3	STK38L
HERC2	SYNE2
ITPR3	TGFA
KRIT1	UQCRC2
LETMD1	VPS13C
LRRC16A	WDR59
MED12	ZC3H7A

**Ingenuity Canonical Pathways**
Coenzyme A Biosynthesis
N-acetylglucosamine Degradation I
Phospholipases
Endothelin-1 Signaling
N-acetylglucosamine Degradation II
Role of BRCA1 in DNA Damage Response
Non-Small Cell Lung Cancer Signaling
Phospholipase C Signaling
Antioxidant Action of Vitamin C
Pancreatic Adenocarcinoma Signaling

**Upstream Transcriptional Regulator**
FOXH1
CITED1
Ncoa6
GSX2
BHLHA15
FOS
VAV2

Supplementary Tables S10. Cluster 6, Genes and top 10 canonical pathways and regulators.

**Genes**
BRIP1
ESCO2

**Ingenuity Canonical Pathways**
Role of BRCA1 in DNA Damage Response

**Upstream Transcriptional Regulator**
CCND1
UXT
FOXM1
IRF1
TCF4
TCF3
FOXO1

Supplementary Tables S11 Cluster 7, Genes and top 10 canonical pathways and regulators.

**Genes**	
ABCA1	LAMA2
AEBP1	LGALS9
AGRN	LYPD1
APOL1	LYVE1
BNC1	MELTF
CARD11	MGP
CCDC3	MYOM3
CDH11	NEAT1
CFH	NEFH
CLDN11	PCDH10
CSF2RB	PCDH17
CTHRC1	PLCB2
CX3CL1	PLCD1
DDR2	PLCL1
DGKA	POSTN
DKK2	PSMB9
DOCK5	PTPRU
DOCK8	SAT1
DPP4	SELENBP1
ERAP1	SEMA7A
FAM129A	SLITRK4
FRAS1	ST6GALNAC1
GATSL3	STXBP2
HSPA12B	TACSTD2
IGFBP5	TFAP2A
IL18R1	TRPV2
ITGB3	TUSC3
KLF4	VCAM1
KRT19	

**Ingenuity Canonical Pathways**
Adrenomedullin signaling pathway
Role of Macrophages, Fibroblasts and Endothelial Cells in Rheumatoid Arthritis
Antioxidant Action of Vitamin C
P2Y Purigenic Receptor Signaling Pathway
Phospholipases
Wnt/Ca+ pathway
Melatonin Signaling
GPCR-Mediated Integration of Enteroendocrine Signaling Exemplified by an L Cell
Aldosterone Signaling in Epithelial Cells
Agrin Interactions at Neuromuscular Junction

**Upstream Transcriptional Regulator**
HTT
NKX2-3
HOXA10
CREB1
SMARCA4
MECP2
STAT1
FOXF1
IRF2
ATF4

Supplementary Tables S12. Cluster 8, Genes and top 10 canonical pathways and regulators.

**Genes**	HSPG2
A2M	IGFBP2
ABCA7	IL17RD
ACE	IL33
ACP5	ITGA10
ADCY4	ITPR1
ANK1	KCND1
ANK3	KCNN4
APLNR	LIMCH1
APOB	LPL
APOL4	LRP1
AQP1	MAMDC2
AQP3	MAN1C1
ARHGEF9	MAN2B2
ATHL1	MFAP2
ATP2A3	MMP17
B3GNT9	MYO18A
BTN3A3	NPR1
C10orf10	PBX1
C10orf128	PCMTD1
C10orf54	PEG10
C14orf132	PIDD1
C1RL	PIK3R3
CALCOCO1	PLPP3
CCND2	PPARGC1B
CD24	PPP1R14A
CD40	PTGIS
CECR1	RAPGEF5
CFI	RP1-152L7.5
CKB	RRAGB
CMKLR1	SCUBE3
COL17A1	SEMA3G
COL1A2	SGCE
COL4A5	SIAE
COLEC12	SIDT2
DPYSL4	SLC16A14
DYSF	SLC46A3
EDA2R	SLCO2A1
ENTPD1	SLCO2B1
EPS8L2	SNED1
ERV3-1	SORT1
FBLN2	STAB1
FBN1	SULF1
FKBP9	TMOD1
GAA	TNFRSF14
GAL3ST4	TNS1
GJA4	TPCN1

**Ingenuity Canonical Pathways**
Atherosclerosis Signaling
Inhibition of Matrix Metalloproteases
Adrenomedullin signaling pathway
Gap Junction Signaling
GP6 Signaling Pathway
eNOS Signaling
Hepatic Fibrosis / Hepatic Stellate Cell Activation
Renin-Angiotensin Signaling
Breast Cancer Regulation by Stathmin1
Dopamine-DARPP32 Feedback in cAMP Signaling

**Upstream Transcriptional Regulator**
KLF2
TP53
SP1
TCF4
CALR
CEBPA
BRD7
ETS1
IKZF1
ASB9

GJA5	TRIM66
GPR146	USHBP1
HEG1	VASH1
HHAT	VCAN
HMCN1	ZNF366

Supplementary Tables S13. AdOSGIN1 vs AdControl, Genes and top 20 canonical pathways and regulators.

**Genes**				
A2M	CMKLR1	HSPA1B	OSGIN2	SLITRK4
ABCA1	COL17A1	HSPA4	P4HA1	SNED1
ABCA7	COL1A2	HSPA6	PACS1	SNRPA1
ACE	COL4A5	HSPB8	PAN2	SORT1
ACP5	COLEC12	HSPD1	PBX1	ST6GALNAC1
ADCY4	CPNE5	HSPG2	PCDH10	STAB1
ADM	CRYAB	IGFBP2	PCDH17	STK38L
AEBP1	CSF2RB	IGFBP5	PCMTD1	STXBP2
AGRN	CTD-2015G9.2	IL17RD	PCMTD2	SULF1
ALDH1A3	CTHRC1	IL18R1	PDE4B	SYNE2
AMDHD2	CX3CL1	IL33	PEG10	TACSTD2
ANGPT2	DDR2	INA	PIDD1	TBXAS1
ANGPTL4	DGKA	ITGA10	PIK3R3	TFAP2A
ANK1	DHRS1	ITGB3	PLA2G4C	TGFA
ANK3	DKK2	ITPR1	PLCB2	TKT
ANKRD33B	DOCK5	ITPR3	PLCD1	TMEM156
AP3M2	DOCK8	JMJD6	PLCL1	TMOD1
APLNR	DPP4	KCND1	PLPP2	TNFRSF14
APOB	DPYSL4	KCNN4	PLPP3	TNFSF18
APOL1	DYSF	KLF4	POSTN	TNS1
APOL4	EDA2R	KRT19	PPARGC1B	TPCN1
AQP1	ENTPD1	LAMA2	PPP1R14A	TRIM16
AQP3	EPS8L2	LENG9	PRDX1	TRIM16L
ARHGEF9	ERAP1	LETMD1	PRDX6	TRIM66
ATHL1	ERV3-1	LGALS9	PSMB9	TRPV2
ATP2A3	EVL	LIMCH1	PTGES3	TUSC3
B3GNT9	F2RL2	LPL	PTGIS	UPP1
BNC1	FAM129A	LRP1	PTPRU	UQCRC2
BTN3A3	FBLN2	LRRC16A	RAPGEF5	USHBP1

**Ingenuity Canonical Pathways**
Aldosterone Signaling in Epithelial Cells
Adrenomedullin signaling pathway
Atherosclerosis Signaling
eNOS Signaling
Hepatic Fibrosis / Hepatic Stellate Cell Activation
Sperm Motility
Cellular Effects of Sildenafil (Viagra)
Dopamine-DARPP32 Feedback in cAMP Signaling
Gap Junction Signaling
Prostanoid Biosynthesis
Endothelin-1 Signaling
GP6 Signaling Pathway
GPCR-Mediated Integration of Enteroendocrine Signaling Exemplified by an L Cell
Synaptic Long Term Depression
Neuropathic Pain Signaling In Dorsal Horn Neurons
Dendritic Cell Maturation
Phospholipases
Inhibition of Matrix Metalloproteases
Thrombin Signaling
Protein Ubiquitination Pathway

**Upstream Transcriptional Regulator**
TP53
EPAS1
HIF1A
KLF2
NKX2-3
NEUROG1
SP1
NFKBIA
ETS1
HTT
STAT3
NUPR1
TWIST1
MAFG
MYB
KEAP1
KDM3A
BACH1
NFE2L2
HOXA10

C10orf10	FBN1	LYPD1	RCBTB1	VASH1
C10orf128	FKBP4	LYVE1	RNF123	VCAM1
C10orf54	FKBP9	MAMDC2	RP1-152L7.5	VCAN
C14orf132	FRAS1	MAN1C1	RPSAP58	VPS13C
C1RL	GAA	MAN2B2	RRAGB	XPOT
CACYBP	GAL3ST4	ME1	SAT1	ZNF366
CALCOCO1	GATSL3	MELTF	SCUBE3	
CAPN11	GCLM	MFAP2	SEC61G	
CARD11	GDF15	MFSD11	SELENBP1	
CCDC3	GJA4	MGP	SELO	
CCND2	GJA5	MLLT11	SEMA3G	
CD24	GPR146	MMP17	SEMA7A	
CD40	HEG1	MT-CYB	SGCE	
CDH11	HHAT	MYO18A	SIAE	
CECR1	HMCN1	MYOM3	SIDT2	
CFH	HMOX1	NEAT1	SLC16A14	
CFI	HOXA9	NEFH	SLC17A9	
CHORDC1	HOXB7	NPR1	SLC35E2B	
CKB	HSP90AA1	NQO1	SLC46A3	
CLDN11	HSPA12B	NUP160	SLCO2A1	
CLIP4	HSPA1A	OSGIN1	SLCO2B1	

Supplementary Tables S14. AdOSGIN2 vs AdControl, Genes and top 20 canonical pathways and regulators.

**Genes**
HERC6
HSPA6
IFIT1
IFIT3
MX2
OASL
OSGIN2
RSAD2
XAF1

**Ingenuity Canonical Pathways**
Interferon Signaling
Role of Lipids/Lipid Rafts in the Pathogenesis of Influenza
Unfolded protein response
Aldosterone Signaling in Epithelial Cells
eNOS Signaling
Huntington's Disease Signaling
Protein Ubiquitination Pathway
Glucocorticoid Receptor Signaling

**Upstream Transcriptional Regulator**
IRF7
STAT3
STAT1
IRF1
IRF5
IRF3
STAT2
SPI1
TRIM24
NFATC2
IRF9
MSC
BRCA1
SP100
IKZF3
SIRT1
CNOT7
SQSTM1
POU2AF1
HOXD10

Supplementary Tables S15. AdOSGIN1&2 vs AdControl, Genes and top 20 canonical pathways and regulators.

Genes			
AHSA1	GCLM	NUP107	TRIM16
ALDH3A2	GDF15	OAS1	TRIM16L
AMDHD2	HAS3	OAS2	TRIM21
ANAPC5	HERC2	OASL	TRIM26
ANGPT2	HERC6	OSGIN1	TRIM69
ANKRD10	HIF3A	OSGIN2	TSPYL2
AP3M2	HIST2H2BE	P4HA1	UBC
APOL6	HMOX1	P4HA2	UBE2L6
ATF3	HSP90AA1	PACS1	UQCRC2
ATXN2	HSP90AB1	PARP14	UTP3
B3GAT3	HSPA1A	PARP9	VPS13C
BAG3	HSPA1B	PATL1	WDR59
BANF1	HSPA4	PAXBP1	XAF1
BATF2	HSPA6	PIGQ	ZC3H7A
BRIP1	HSPA8	PLAUR	ZDHHC6
C19orf66	HSPB1	PLD1	ZFAND2A
C22orf29	HSPB8	PLEKHB2	ZNF207
C3orf52	HSPD1	PLSCR1	ZNF622
CACYBP	HSPE1	PMAIP1	
CBARP	HSPH1	PPCS	
CHD1L	IER5L	PPP1R18	
CHKA	IFI35	PRDX1	
CHORDC1	IFI44L	PRPF38B	
CLIP4	IFIH1	PTGES3	
CPNE8	IFIT1	RCBTB1	
CRYAB	IFIT2	RFC3	
CRYZ	IFIT3	RNF123	
DDX58	IFIT5	RNF19B	
DDX60	IFITM1	RSAD2	
DDX60L	IL7R	RSRP1	
DEDD2	IRF7	SAMD9	

**Ingenuity Canonical Pathways**
Aldosterone Signaling in Epithelial Cells
Protein Ubiquitination Pathway
Interferon Signaling
NRF2-mediated Oxidative Stress Response
Activation of IRF by Cytosolic Pattern Recognition Receptors
Unfolded protein response
Role of Pattern Recognition Receptors in Recognition of Bacteria and Viruses
Role of RIG1-like Receptors in Antiviral Innate Immunity
eNOS Signaling
Glucocorticoid Receptor Signaling
Aryl Hydrocarbon Receptor Signaling
Pathogenesis of Multiple Sclerosis
Hypoxia Signaling in the Cardiovascular System
Huntington's Disease Signaling
Choline Biosynthesis III
Primary Immunodeficiency Signaling
Xenobiotic Metabolism Signaling
Mitotic Roles of Polo-Like Kinase
IL-17A Signaling in Gastric Cells
Coenzyme A Biosynthesis

**Upstream Transcriptional Regulator**
IRF7
HSF1
NKX2-3
STAT1
IRF5
IRF1
CNOT7
TRIM24
IRF3
STAT2
STAT3
IRF9
SPI1
PML
BRCA1
SIRT1
IRF2
NFE2L2
FBXW7
TP53

DFNA5	ISG15	SAMD9L	
DHRS1	ITPR3	SBDSP1	
DHRS11	JMJD6	SEC61G	
DHX58	KRIT1	SELO	
DNAJA1	LGALS8	SERPINH1	
DNAJA4	LUC7L3	SESTD1	
DNAJB1	LY6E	SLC15A3	
DNAJB6	MED12	SLC35E2B	
DTX3L	MFSD11	SLC37A1	
EEF2K	MICB	SMARCC2	
EIF2AK2	MLKL	SNX3	
EPSTI1	MLLT11	SP110	
ESCO2	MRPL18	SPATS2L	
EVI2B	MSX1	SQSTM1	
EVL	MX1	STIP1	
FKBP4	MX2	SYNE2	
FTL	NAPG	TAP1	
GBP1	NDRG1	TGFA	
GCH1	NQO1	TKT	

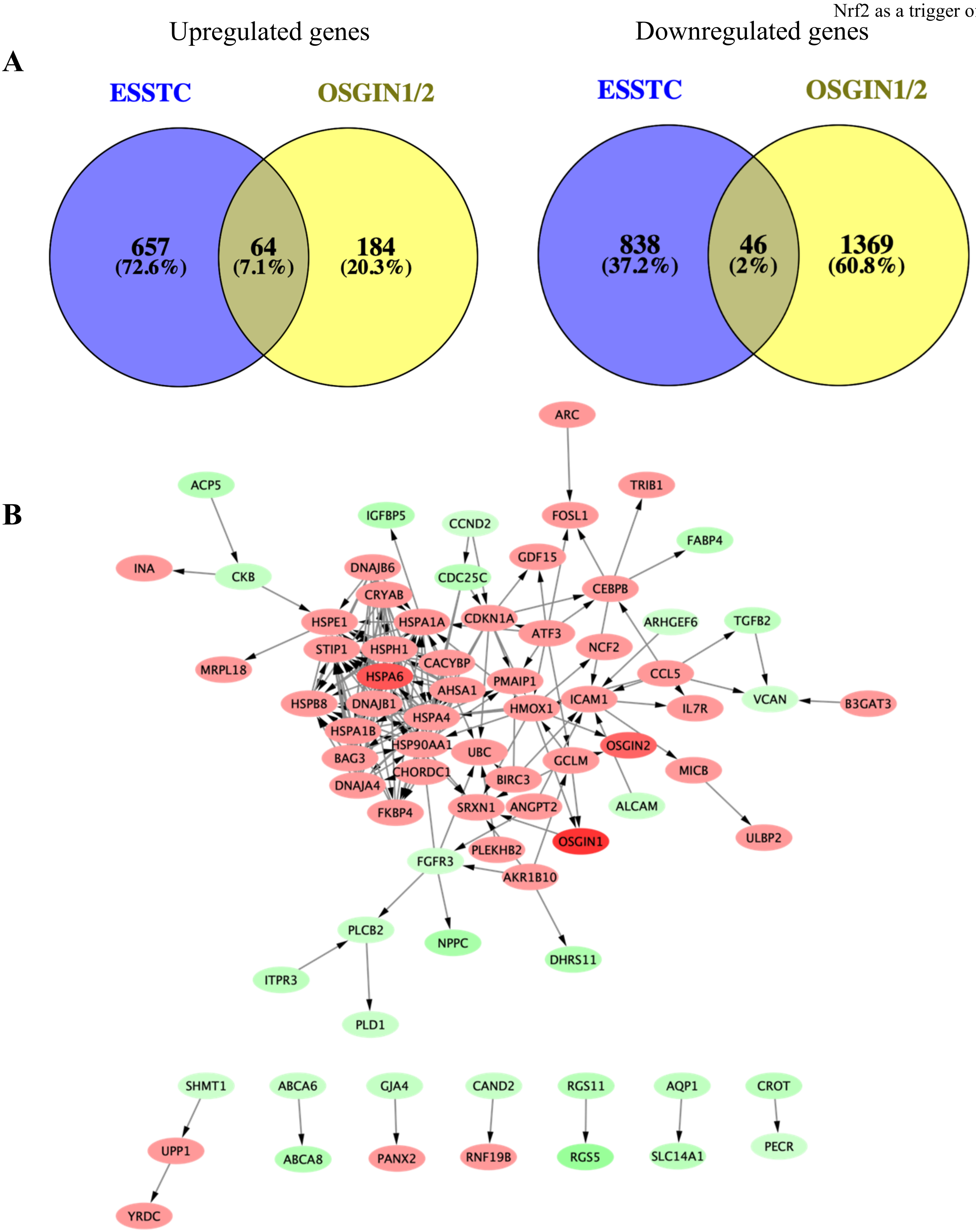

Figure S14. ESSTC and OSGIN1&2 coregulated genes: a) Genes significantly regulated under conditions of ESSTC (compared to ESS >2-fold change, p adj <0.05) and OSGIN1/2 (compared to virus control, >1.5-fold change, p adj < 0.05). B) neighbor network analysis was carried out using cytoscape. ATF3 was observed to be the gene regulator of GDF15 and HSP70 (HSPA1A).

Supplementary Tables S16, Genes significantly regulated under conditions of ESSTC (compared to ESS >2 -fold change, p adj <0.05) and OSGIN1/2 (compared to virus control, >1.5-fold change, p adj < 0.05)

Upregulated Genes

HSPA6	GDF15	UPP1	INA
ATF3	KLHL21	GCLM	ICAM1
CRYAB	OSGIN2	HSPB8	CCL5
HSPA7	BAMBI	BAG3	HSP90AA1
MMP10	SLC12A8	TMEM140	YRDC
BIRC3	HSPA1A	DEDD2	ARC
IL7R	TRIB1	TNIP3	CHORDC1
ANGPT2	HMOX1	JMJD6	HSPA4
SRXN1	NCF2	FOSL1	B3GAT3
ZFAND2A	MICB	PANX2	HSPE1
PMAIP1	MSX1	STIP1	TMEM156
DNAJA4	UBC	FKBP4	DNAJB6
AKR1B10	TSPYL2	AHSA1	CACYBP
OSGIN1	HSPA1B	HSPH1	MRPL18
CDKN1A	CEBPB	SPATS2L	PLEKHB2
DNAJB1	FAM46A	RNF19B	ULBP2

Downregulated genes

TGFB2	ABCA8	ADCY4	GJA4
PECR	VCAN	OPRL1	IGFBP5
CDC25C	ALCAM	CKB	CAPN11
ANKRD35	PLD1	EEF2K	MAMDC2
ANXA2P1	LIMCH1	NPPC	VIPR1
PLCB2	PALM	EVL	ACP5
SHMT1	ARHGEF6	ACAD11	GJA4
PIK3IP1	FABP4	AQP1	IGFBP5
DHRS11	CROT	CCND2	CAPN11
DDR2	CAND2	APLN	MAMDC2
RGS5	ABCA6	CAPN11	VIPR1
ABCA7	ASAP3	MAMDC2	ACP5
ITPR3	SLC14A1	VIPR1	GJA4
GRB14	LYPD1	ACP5	IGFBP5
RGS11	FGFR3		

A

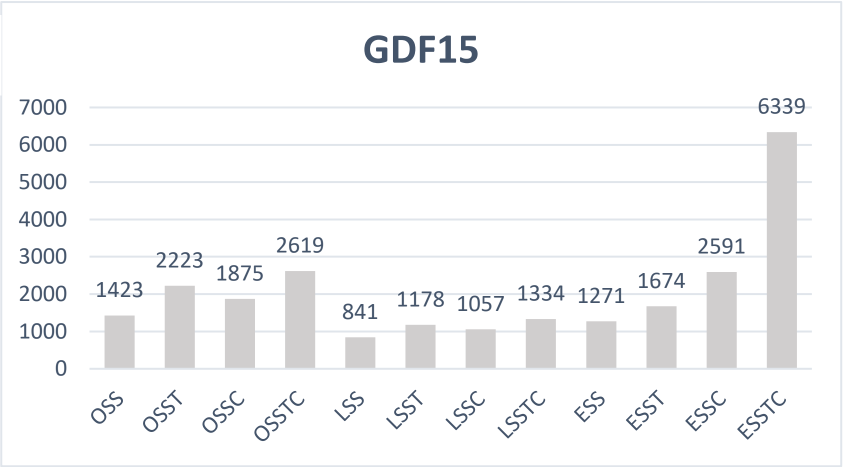

B

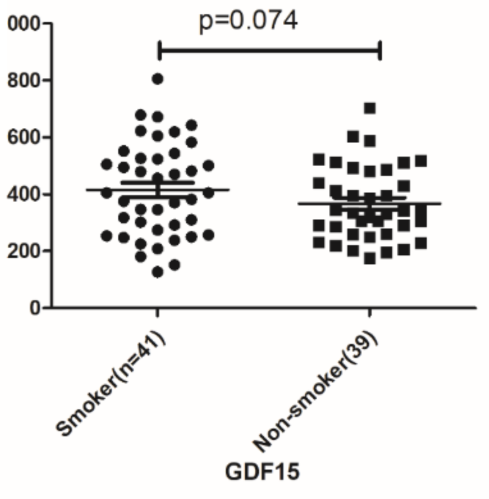

Figure S15, GFD15 regulation. A) Transcriptomic evaluation of GDF15 in HCAECs cultured under oscillatory (OSS), laminar (LSS) and elevated (ESS) shear stress on HCAECs treated with control, 3 doses of 5ng/ml TNFα (T), 3 doses of 10%CSE (C) or the combination of TNFα and CSE (TC), n=3 per condition, as described in Fig 1A. ESSTC significantly increased compared to ESS control (adj p = 1.2E-06). B) comparison of the effect of smoking on GDF15 levels in ACS patient serum combining both plaque rupture and plaque erosion patients.

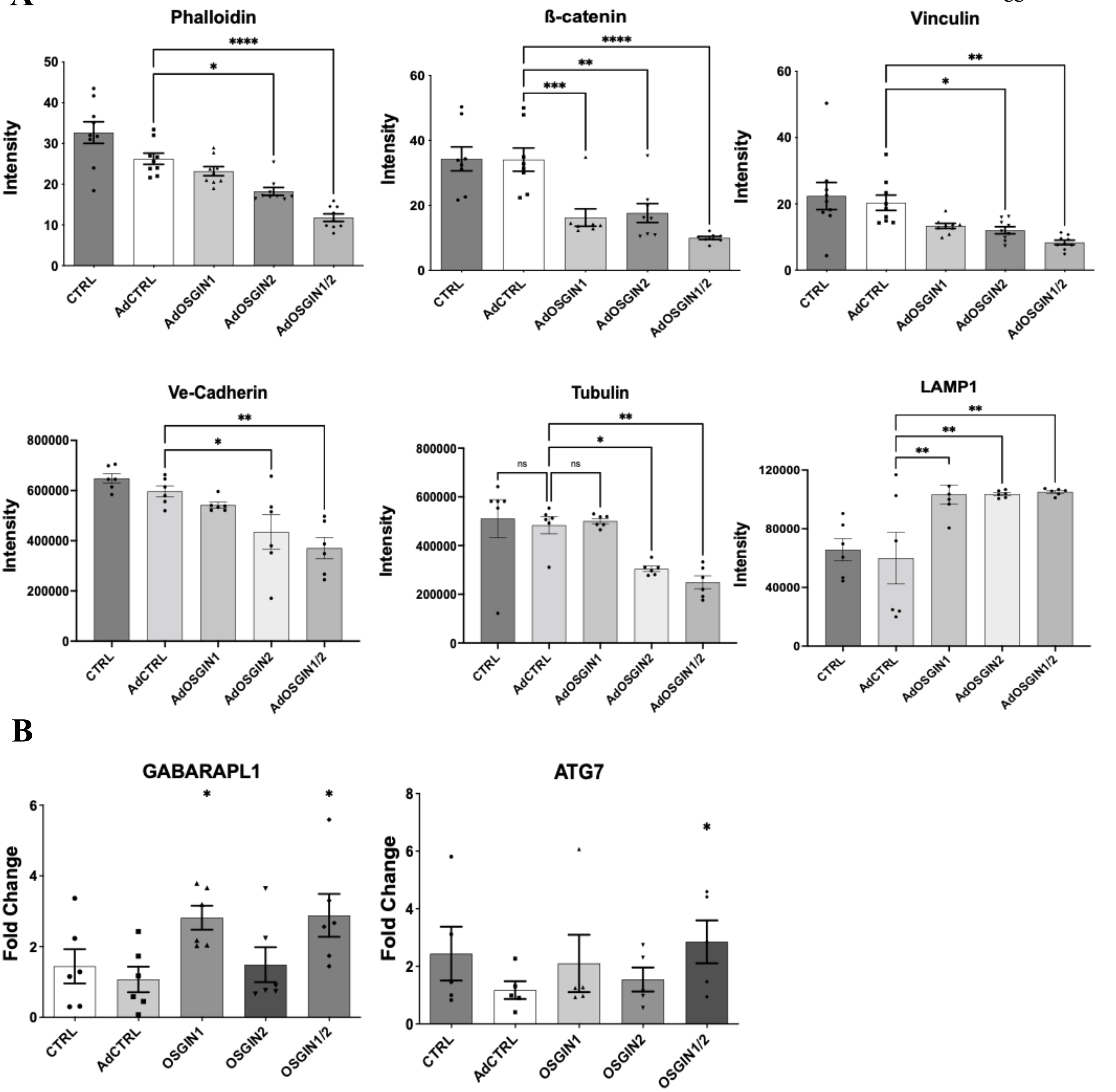

Figure S16: a) Immunocytochemical analysis indicated loss of focal adhesions, stress fibres and tubulin (n=3; P<0.05, P<0.01 and P<0.001), LAMP1 accumulation around nuclei was reported. b) GABARAPL1 (in AdOSGIN1 and AdOSGIN1+2) and ATG7 (AdOSGIN1+2) gene expression level increased compared to AdCTRL *P<0.05.

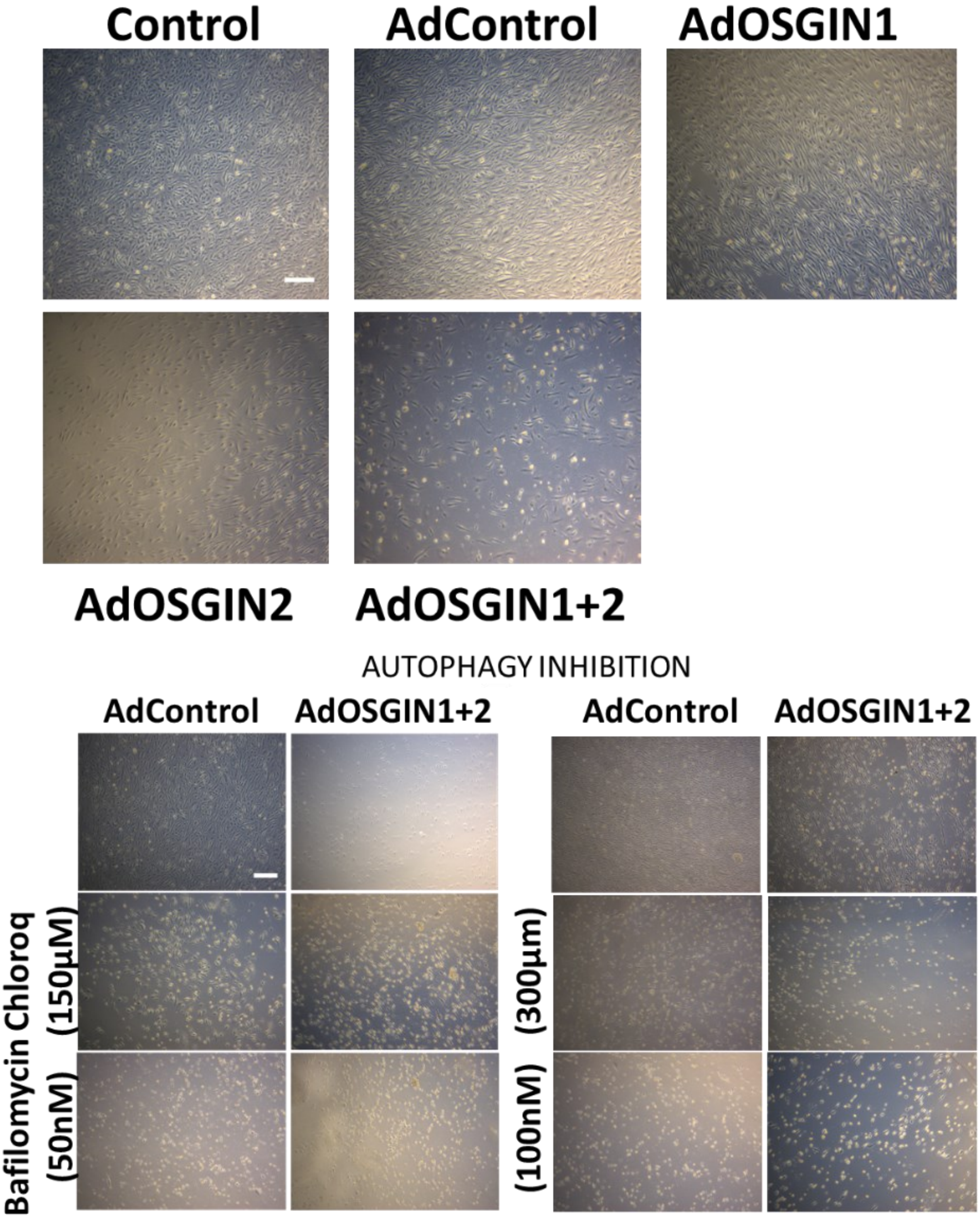

Figure S17. Images of HCAECs treated with chloroquine (150μM), bafilomycin (50nM), or OSGIN1&2 overexpression induced comparable, non-synergistic detachment (mean ± SD, two-way ANOVA, **P<0.01, ***P<0.001 v AdCTRL, n=4) corresponding to the analysis presented in Fig 7C in the main text.

RESCUE EXPERIMENT

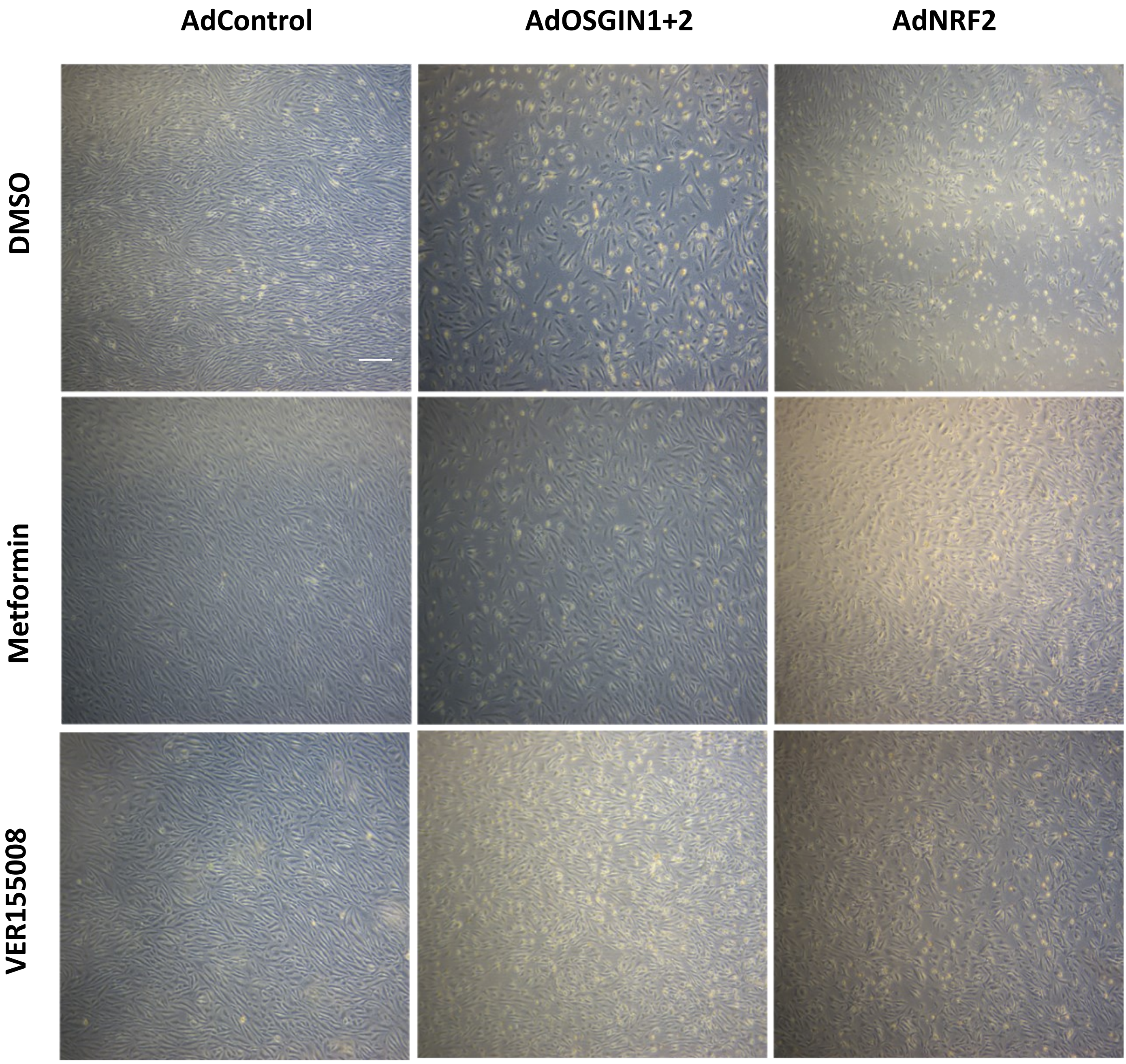

Figure S18: Confluent layer of ECs was seeded on a six-well plate. Adenoviral overexpression (AdCTRL, AdOSGIN1+2, and AdNRF2 was carried out. Following adenoviral transfection, EC were flowed on orbital shaker (210rpm, 3ml) in combination with DMSO, Metformin, and VER-155008) for 72hrs. (See quantification Figure 5E main text)

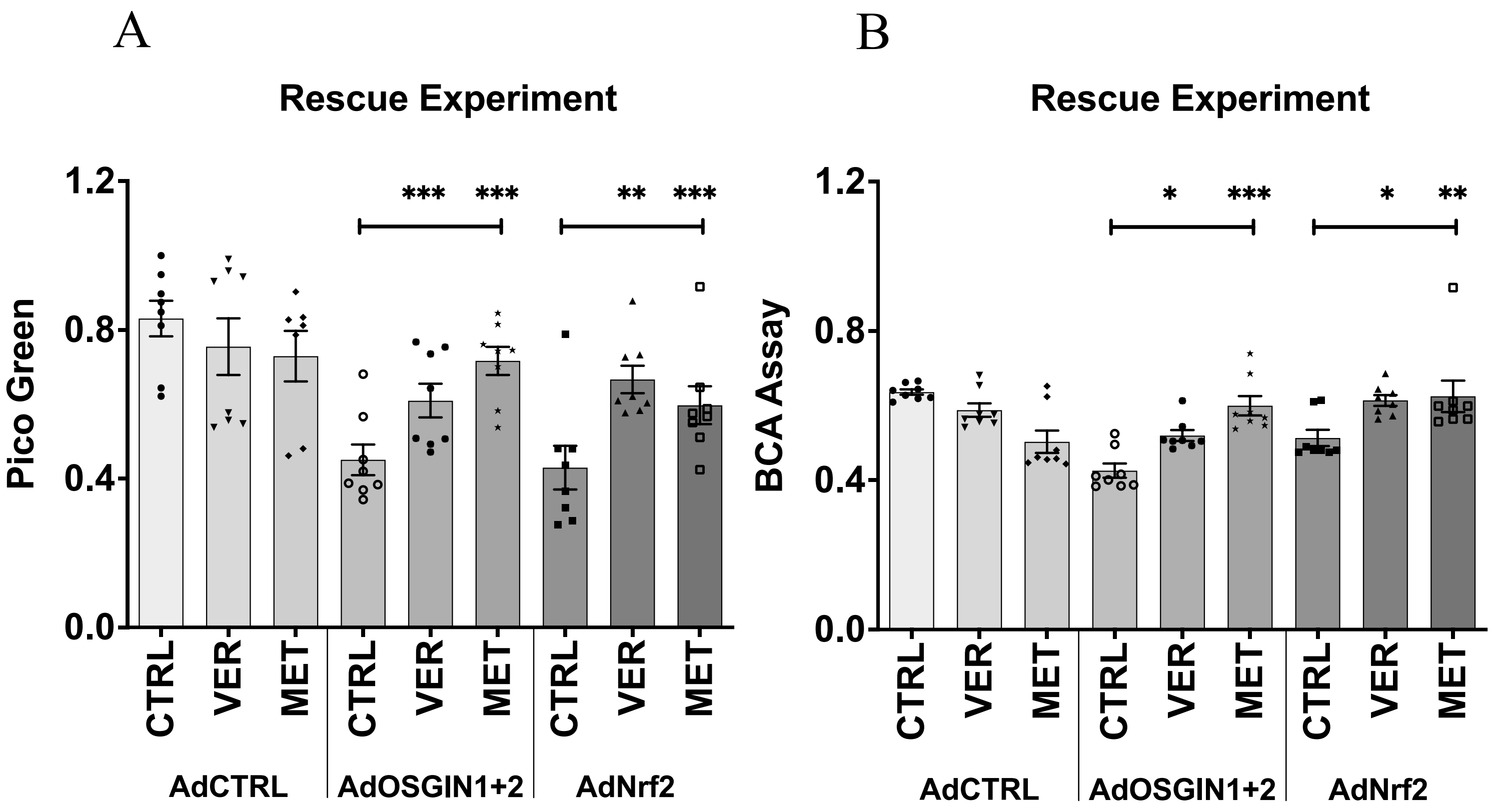

Figure S19. Comparison of different normalisation techniques. Because of the change in cell size, depth (and therefore assumed volume) and increase in number of nuclei per cell (Figures S14c,d; S15; S16), we calculated cell detachment as % coverage (Figure 5E main text). We repeated the analysis of detachment using by A) DNA quantification [2, 16, 17] or B) protein content of the cell lysate. These gave equivalent result as presented in Figure 5E. OSGIN1+2 or Nrf2-mediated cell detachment was reduced by co-treatment with Ver155008 (15μM), or Metformin (100μM); (*$P<0.05$, **$P<0.01$, ***$P<0.001$, n=3, Two-way ANOVA).

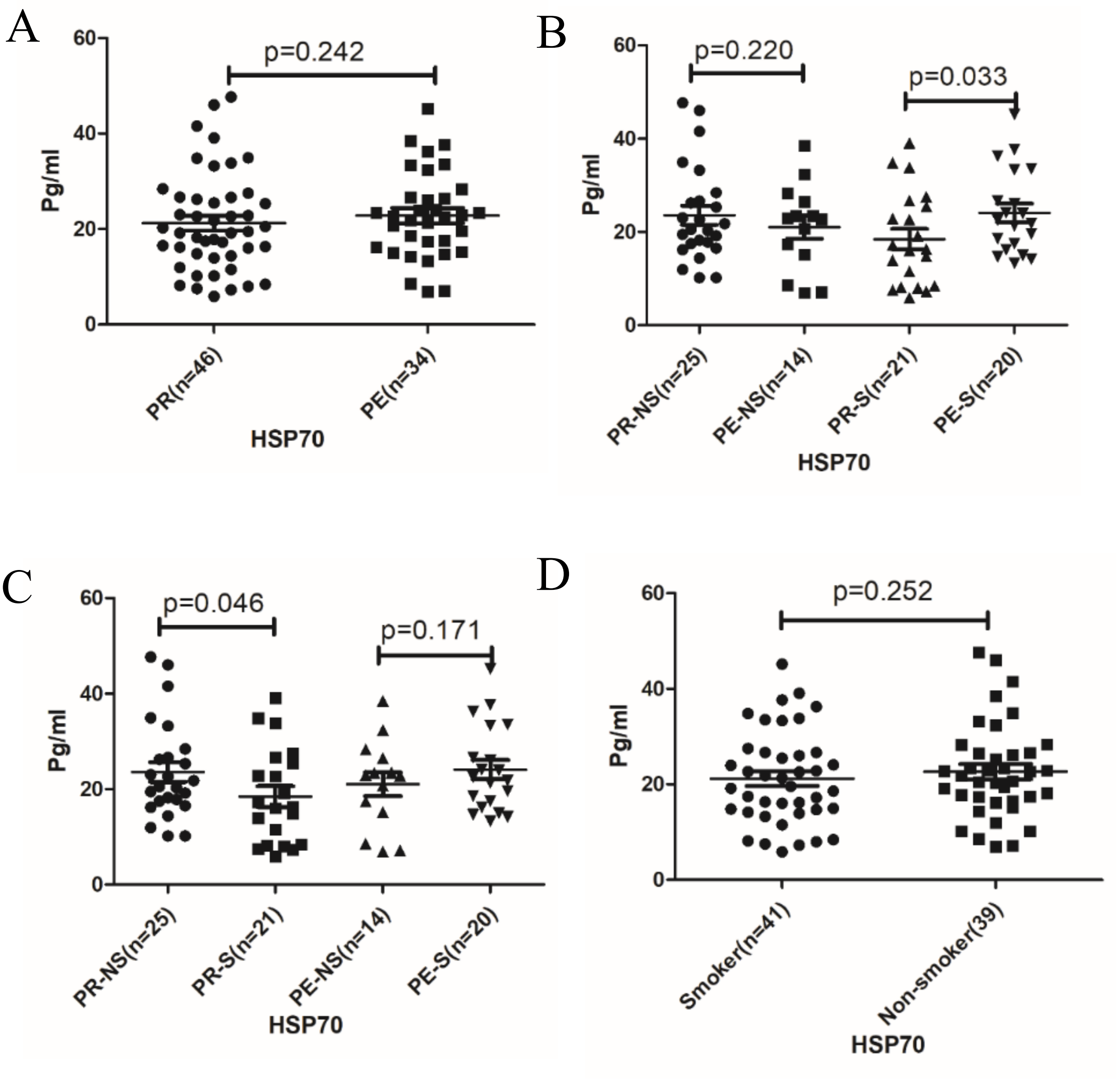

Figure S20. HSP70 quantification in serum of ACS serum. A) No significant differences were observed between patients with OCT-defined plaque rupture (PR) and plaque erosion (PE). B) subgroup analysis of PR and PE in non-smokers (NS) and smokers (S), indicating a significant difference between PR and PE only in smokers. C) comparison between smokers and non-smokers between PR and PE groups didn't observe a significant interaction. D) There was no overall effect of smoking on HSP70 levels in ACS patients.

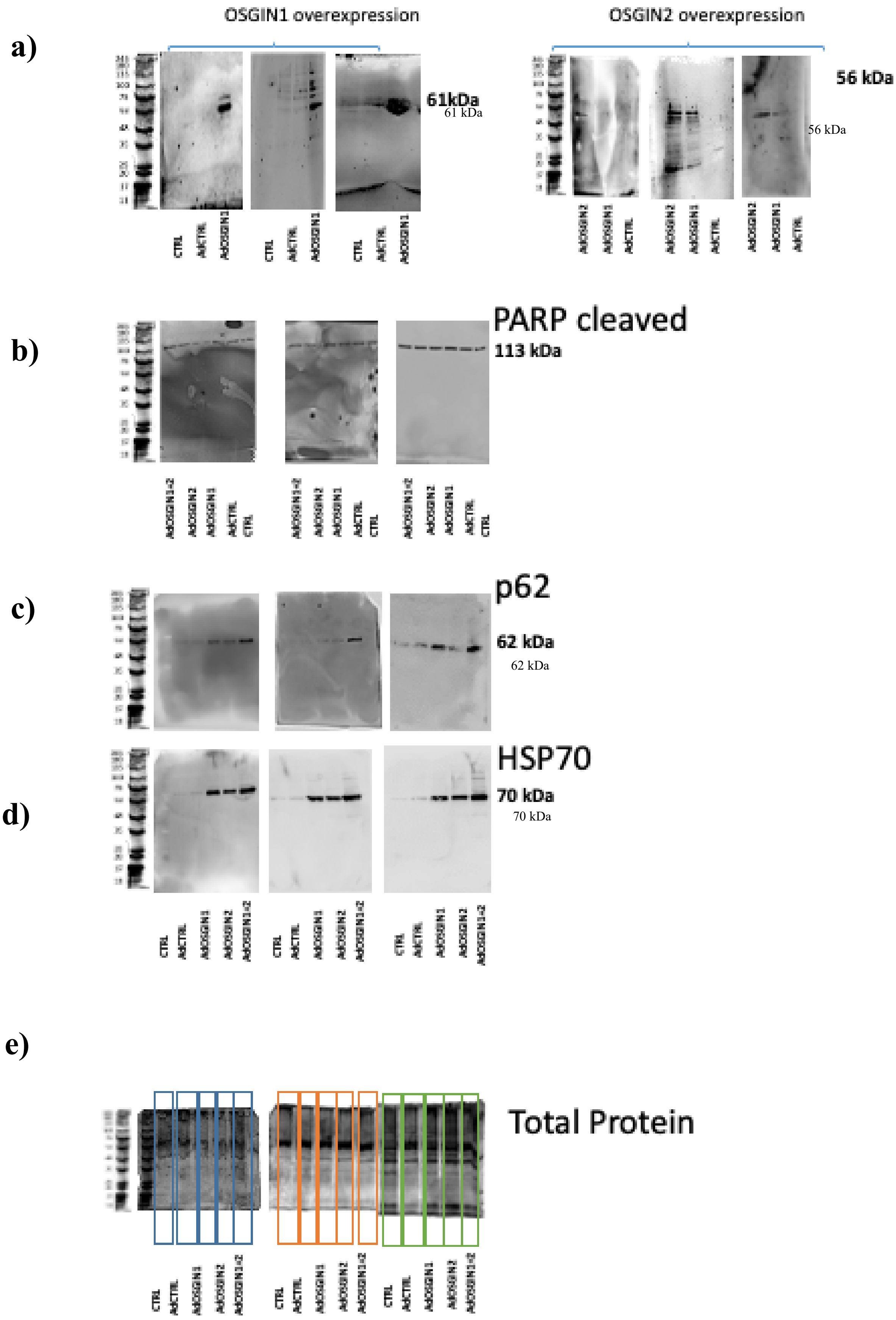
a)
OSGIN1 overexpression
OSGIN2 overexpression
61kDa
61 kDa
56 kDa
56 kDa
b)
PARP cleaved
113 kDa
c)
p62
62 kDa
62 kDa
d)
HSP70
70 kDa
70 kDa
e)
Total Protein

Figure S21: a) Overexpression of OSGINs was evaluated through western blotting. b) PARP cleavage antibody didn’t show any cleave of the PARP protein confirming it was no apoptotic pathway related. c) Total protein (e) quantification was used to determine p62 and HSP70 accumulation

a)

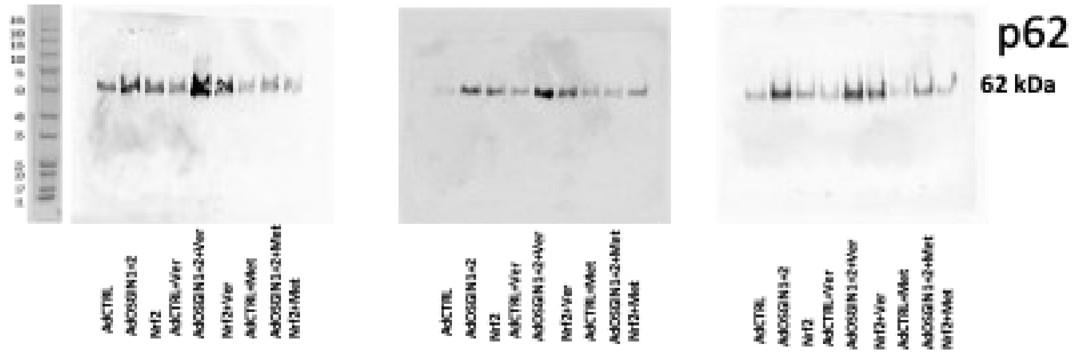
p62
62 kDa
AdCTRL
AdOSGIN1+2
Nrf2
AdCTRL+Ver
AdOSGIN1+2+Ver
Nrf2+Ver
AdCTRL+Met
AdOSGIN1+2+Met
Nrf2+Met

b)

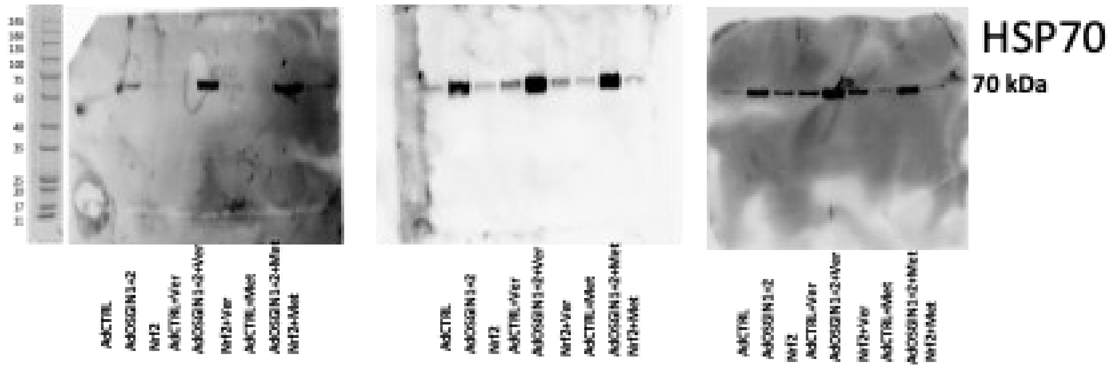
HSP70
70 kDa
AdCTRL
AdOSGIN1+2
Nrf2
AdCTRL+Ver
AdOSGIN1+2+Ver
Nrf2+Ver
AdCTRL+Met
AdOSGIN1+2+Met
Nrf2+Met

c)

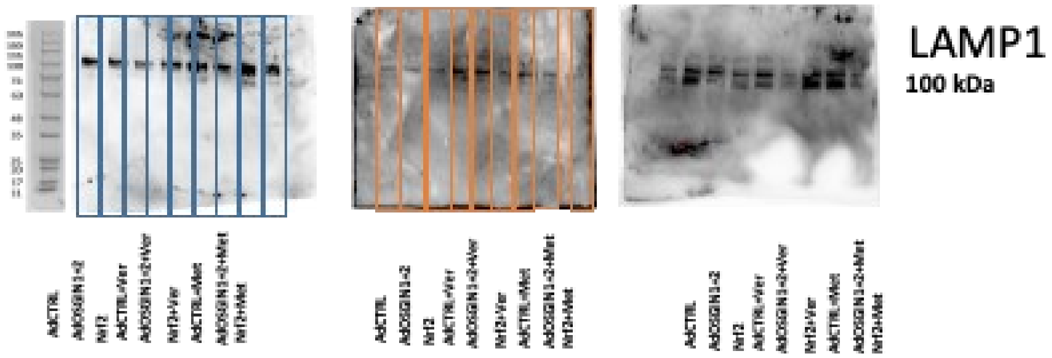
LAMP1
100 kDa
AdCTRL
AdOSGIN1+2
Nrf2
AdCTRL+Ver
AdOSGIN1+2+Ver
Nrf2+Ver
AdCTRL+Met
AdOSGIN1+2+Met
Nrf2+Met

d)

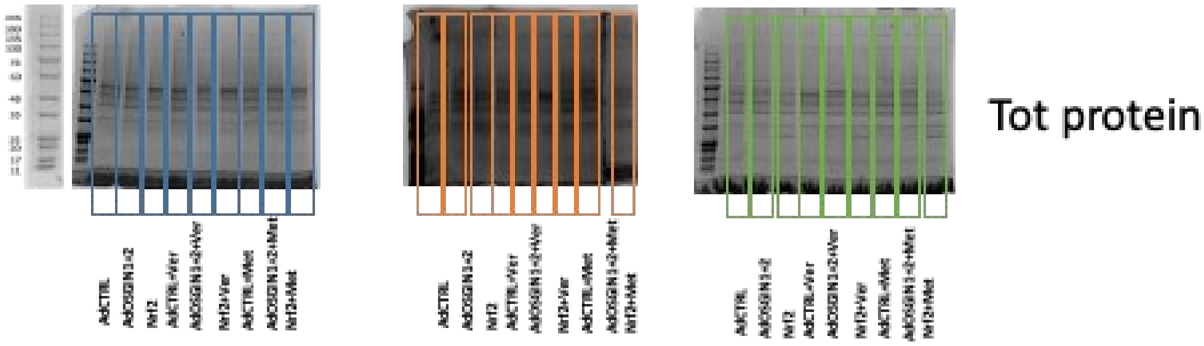
Tot protein
AdCTRL
AdOSGIN1+2
Nrf2
AdCTRL+Ver
AdOSGIN1+2+Ver
Nrf2+Ver
AdCTRL+Met
AdOSGIN1+2+Met
Nrf2+Met

Figure S22: Rescue experiment was carried on orbital shaker using Metformin and VER155008. Following orbital shaker experiment ECs were lysate and total protein (d) quantification was used to determine (a)p62, (b)HSP70 and (c)LAMP1(western blotting analysis).

References

1. Warboys CM, Berson RE, Mann GE, Pearson JD, Weinberg PD. Acute and chronic exposure to shear stress have opposite effects on endothelial permeability to macromolecules. *Am. J. Physiol.-Heart Circul. Physiol.* 2010;298:H1850-H1856
2. Hazell GGJ, Peachey AMG, Teasdale JE, Sala-Newby GB, Angelini GD, Newby AC, White SJ. Pi16 is a shear stress and inflammation-regulated inhibitor of mmp2. *Scientific reports*. 2016;6:39553
3. Pelossof R, Fairchild L, Huang C-H, Widmer C, Sreedharan VT, Sinha N, Lai D-Y, Guan Y, Premsrirut PK, Tschaharganeh DF, Hoffmann T, Thapar V, Xiang Q, Garippa RJ, Rätsch G, Zuber J, Lowe SW, Leslie CS, Fellmann C. Prediction of potent shrnas with a sequential classification algorithm. *Nature Biotechnology*. 2017;35:350-353
4. Betancur J, Yoda M, Tomari Y. Mirna-like duplexes as rnai triggers with improved specificity. *Frontiers in Genetics*. 2012;3
5. Andrews S. Fastqc a quality control tool for high throughput sequence data. 2018;2018
6. Bolger AM, Lohse M, Usadel B. Trimmomatic: A flexible trimmer for illumina sequence data. *Bioinformatics*. 2014;30:2114-2120
7. Dobin A, Davis CA, Schlesinger F, Drenkow J, Zaleski C, Jha S, Batut P, Chaisson M, Gingeras TR. Star: Ultrafast universal rna-seq aligner. *Bioinformatics*. 2013;29:15-21
8. R:CoreTeam. R: A language and environment for statistical computing. *R Foundation for Statistical Computing, Vienna, Austria*. 2014
9. Risso D, Ngai J, Speed TP, Dudoit S. Normalization of rna-seq data using factor analysis of control genes or samples. *Nat Biotechnol*. 2014;32:896-902
10. Love MI, Huber W, Anders S. Moderated estimation of fold change and dispersion for rna-seq data with deseq2. *Genome Biol*. 2014;15:550
11. Warnes GR, Bolker B, Bonebakker L, Gentleman R, Huber W, Liaw A, Lumley T, Maechler M, Magnusson A, Moeller S, Schwartz M, Venables B. Gplots: Various r programming tools for plotting data. 2016
12. Kramer A, Green J, Pollard J, Jr., Tugendreich S. Causal analysis approaches in ingenuity pathway analysis. *Bioinformatics*. 2014;30:523-530
13. Jia H, Abtahian F, Aguirre AD, Lee S, Chia S, Lowe H, Kato K, Yonetsu T, Vergallo R, Hu S, Tian J, Lee H, Park S-J, Jang Y-S, Raffel OC, Mizuno K, Uemura S, Itoh T, Kakuta T, Choi S-Y, Dauerman HL, Prasad A, Toma C, McNulty I, Zhang S, Yu B, Fuster V, Narula J, Virmani R, Jang I-K. In vivo diagnosis of plaque erosion and calcified nodule in patients with acute coronary syndrome by intravascular optical coherence tomography. *Journal of the American College of Cardiology*. 2013;62:10.1016/j.jacc.2013.1005.1071
14. Ajime TT, Serré J, Wüst RCI, Messa GAM, Poffé C, Swaminathan A, Maes K, Janssens W, Troosters T, Degens H, Gayan-Ramirez G. Two weeks of smoking cessation reverse cigarette smoke-induced skeletal muscle atrophy and mitochondrial dysfunction in mice. *Nicotine & Tobacco Research*. 2020
15. Teasdale JE, Newby AC, Timpson NJ, Munafo MR, White SJ. Cigarette smoke but not electronic cigarette aerosol activates a stress response in human coronary artery endothelial cells in culture. *Drug Alcohol Depend.* 2016;163:256-260
16. White S, Hayes E, Lehoux S, Jeremy J, Horrevoets A, Newby A. Characterization of the differential response of endothelial cells exposed to normal and elevated laminar shear stress. *J. Cell. Physiol.* 2011;226:2841 - 2848
17. Teasdale JE, Hazell GGJ, Peachey AMG, Sala-Newby GB, Hindmarch CCT, McKay TR, Bond M, Newby AC, White SJ. Cigarette smoke extract profoundly suppresses tnfα-mediated proinflammatory gene expression through upregulation of atf3 in human coronary artery endothelial cells. *Scientific reports*. 2017;7:39945
